# Temperature-feedback upconversion nanocomposite for accurate photothermal therapy at facile temperature

**DOI:** 10.1038/ncomms10437

**Published:** 2016-02-04

**Authors:** Xingjun Zhu, Wei Feng, Jian Chang, Yan-Wen Tan, Jiachang Li, Min Chen, Yun Sun, Fuyou Li

**Affiliations:** 1 Institutes of Biomedical Sciences, Fudan University, 220 Handan Road, Shanghai 200433, China; 2 Department of Chemistry, State Key Laboratory of Molecular Engineering of Polymers, Fudan University, 220 Handan Road, Shanghai 200433, China; 3 Department of Physics, Fudan University, 220 Handan Road, Shanghai 200433, China; 4 Collaborative Innovation Center of Chemistry for Energy Materials, Fudan University, 220 Handan Road, Shanghai 200433, China

## Abstract

Photothermal therapy (PTT) at present, following the temperature definition for conventional thermal therapy, usually keeps the temperature of lesions at 42–45 °C or even higher. Such high temperature kills cancer cells but also increases the damage of normal tissues near lesions through heat conduction and thus brings about more side effects and inhibits therapeutic accuracy. Here we use temperature-feedback upconversion nanoparticle combined with photothermal material for real-time monitoring of microscopic temperature in PTT. We observe that microscopic temperature of photothermal material upon illumination is high enough to kill cancer cells when the temperature of lesions is still low enough to prevent damage to normal tissue. On the basis of the above phenomenon, we further realize high spatial resolution photothermal ablation of labelled tumour with minimal damage to normal tissues *in vivo*. Our work points to a method for investigating photothermal properties at nanoscale, and for the development of new generation of PTT strategy.

## Abstract (web summary)

In photothermal therapy for cancer treatment, hyperthermic effects can damage healthy tissues and inhibit therapeutic efficacy. Here, the authors use core-shell lanthanide-based nanoparticles to monitor microscopic temperatures and to ablate cancer tissue at low temperatures.

## Graphical abstract

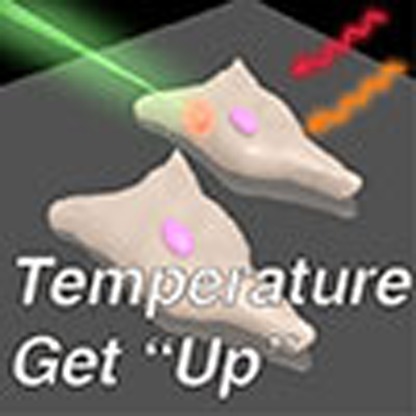

The past few decades have witnessed significant efforts in the treatment of cancer^1^,^2^. Among the existing treatment methods, thermal therapy has become an important treatment modality^3^. Conventional approaches including radiofrequency or microwave ablation have been widely used in clinic^4^,^5^. However, these approaches relying on macroscopic heat sources have a relatively large destruction range causing normal tissue damages and even some serious systemic side effects^6^,^7^. Photothermal therapy (PTT), using photoabsorbing molecules or nanoparticles as microscopic heat sources, is expected to improve the therapeutic accuracy and reduce injury to normal tissues^8^,^9^,^10^,^11^,^12^,^13^. However, the current method to monitor PTT regards the entire lesion containing PTT agents as a macroscopic heat source and keep the overall temperature of the lesion (here defined as apparent temperature) at high level in line with the temperature definition for conventional thermal therapy (usually 42–45 °C)^14^,^15^. In some cases, the temperature is even higher^16^,^17^. Such a high apparent temperature can damage normal tissues adjacent to the lesions due to massive heat transfer, therefore, leading to more side effect and inhibiting the therapeutic accuracy of PTT. In our view, distinguished from traditional thermal therapy methods using macroscopic heat source and requiring apparent temperature as reference, PTT uses heat source at nanoscale, so correspondingly the temperature of those nanoparticles (here defined as the eigen temperature) during photothermal process should be the prerequisite to determine the temperature threshold for effective and minimally harmful PTT. Although some previous studies have referred to the measurement of the eigen temperature of gold nanostructures^18^,^19^, to date, no data were reported to utilize the eigen temperature during PTT in a real biosystem to achieve therapeutic effect with high spatial resolution under facile apparent temperature. It still remains unsolved to seek for an adequate thermal-sensitive system that is stable and not affected by the complex biological condition to report the eigen temperature of photothermal agents, thus determining the temperature threshold for accurate and facile therapy. If there exists a suitable way to monitor the eigen temperature during PTT, then it will not only open a window to learn the temperature of PTT agents at microscopic level, but also innovate the PTT strategy for better therapeutic efficacy.

To monitor the eigen temperature of PTT agents in biological systems, temperature-sensing luminescent materials are appropriate options as the optical signals provide high resolution and sensitivity. A series of luminescent temperature-sensing probes have been developed including organic dyes, polymers, QDs and lanthanide-based upconversion nanophosphors (Ln-UCNPs)^20^,^21^,^22^,^23^,^24^,^25^. Among those, Ln-UCNPs which allow the conversion of lower-energy light in the near-infrared (NIR) region into higher energy emissions have many advantages to be served as imaging or therapy agents such as superior photostability, non-blinking, absence of autofluorescence of biological tissue and low-energy NIR radiation^26^,^27^,^28^,^29^,^30^,^31^,^32^,^33^,^34^,^35^,^36^,^37^,^38^,^39^,^40^,^41^,^42^,^43^. On the basis of the above merits, Ln-UCNPs are ideal probes to real-time sensing the eigen temperature of PTT agents in biological system. Here we build a carbon-coated core-shell upconversion nanocomposite NaLuF_4_:Yb,Er@NaLuF_4_@Carbon (csUCNP@C) to investigate the possible difference between the apparent and eigen temperature and evaluate the therapeutic effect of implementing photothermal therapy at low apparent temperature, but a much higher eigen temperature in real biosystems. Moreover, this kind of nanocomposite can also be served as theranostic agents as the upconversion core and carbon shell endorse it with a good imaging quality and PTT efficacy.

## Results

### Characterization of temperature-feedback csUCNP@C

**Figure 1:**
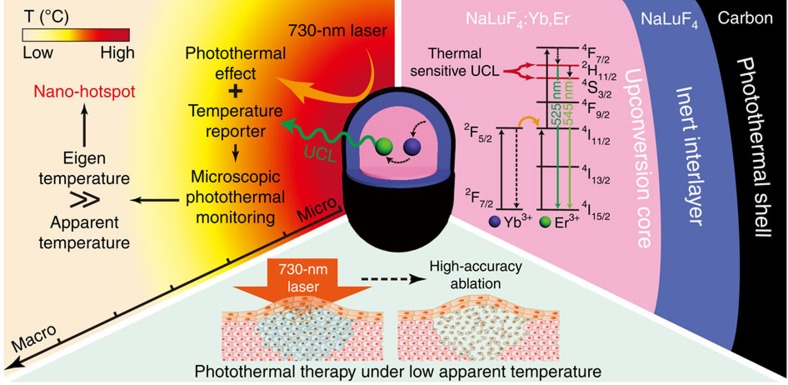
Schematic of csUCNP@C for accurate PTT at facile temperature. The csUCNP@C exhibit both UCL emission and photothermal effect. With temperature-sensitive UCL emission, csUCNP@C was used to monitor the change in microscopic temperature of the photoabsorber (carbon shell) under 730-nm irradiation. The eigen temperature of csUCNP@C was much higher than the apparent temperature observed macroscopically, indicating that csUCNP@C acted as a nano-hotspot at the microscopic level. By utilizing the high eigen temperature during photothermal process, accurate PTT, which prevent the damage to normal tissues can be realized.

**Figure 2:**
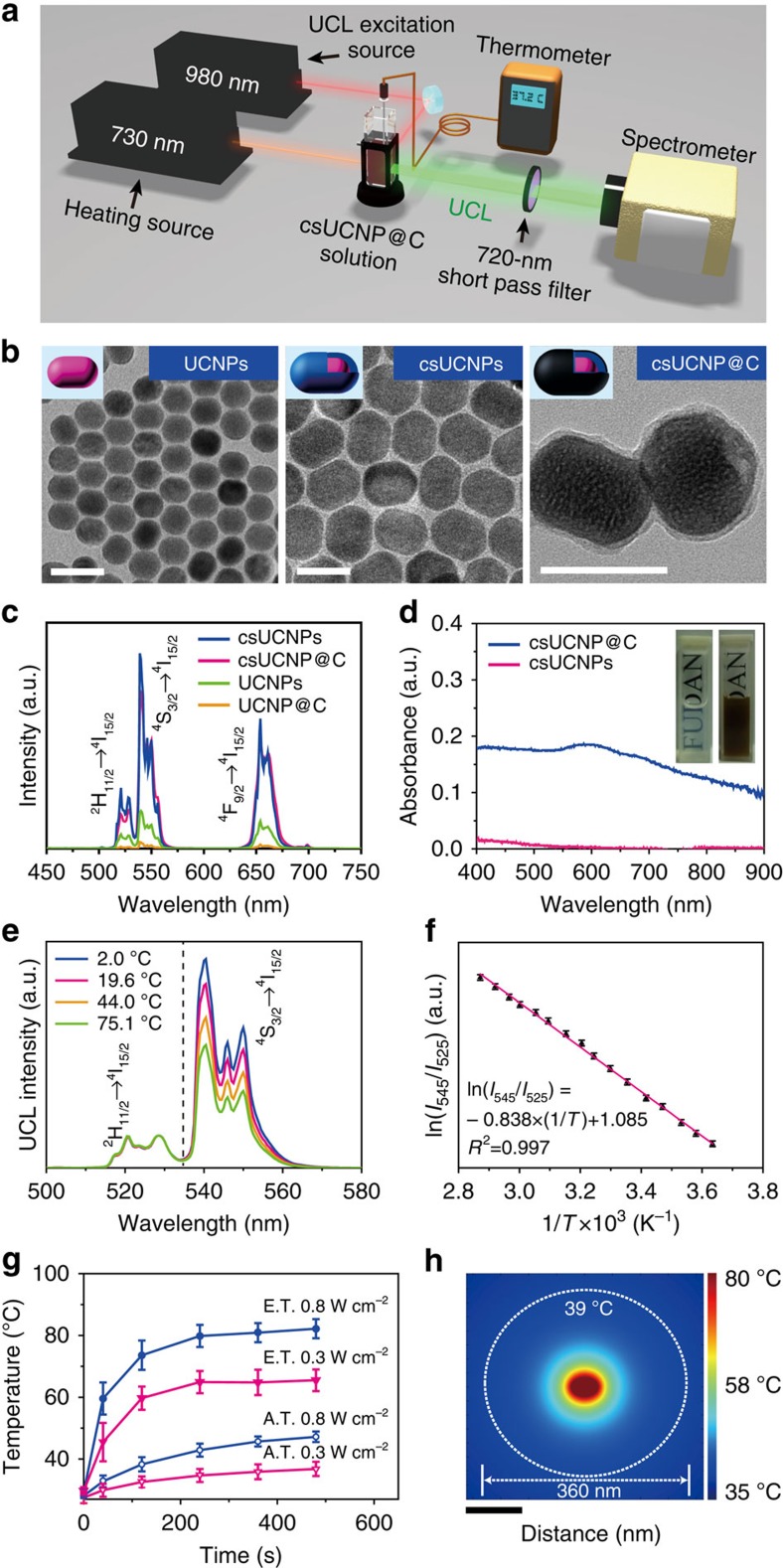
Characterization and temperature-sensing properties of csUCNP@C. (**a**) Schematic diagram of the detection of the eigen temperature of csUCNP@C. (**b**) TEM images of UCNPs (left), csUCNPs (middle) and csUCNP@C (right). Scale bar, 50 nm. (**c**) UCL emission spectra of UCNPs, UCNPs@C, csUCNPs and csUCNP@C in the aqueous dispersion with the same concentration of luminescence centre Er^3+^. (**d**) Powder absorption spectra of csUCNPs and csUCNP@C. (**e**) UCL emission spectra of Er^3+^-doping csUCNP@C at different temperatures by external heating. The peaks were normalized at 528.5 nm. (**f**) A plot of ln(*I*_525_/*I*_545_) versus 1/*T* to calibrate the thermometric scale for csUCNP@C. *I*_525_ and *I*_545_ indicate the UCL emission of the ^2^H_11/2_ → ^4^I_15/2_ and ^4^S_3/2_ → ^4^I_15/2_ transitions, respectively. Average values of *I*_525_/*I*_545_ under different temperature were given to fit the calibration curve based on three times measurements of UCL spectrum. Error bars were defined as s.d. (**g**) Elevation of apparent temperature (A.T.) and eigen temperature (E.T.) of csUCNP@C (1 mg ml^−1^) in aqueous dispersion under irradiation with a 730-nm laser at 0.8 and 0.3 W cm^−2^. Average value of A.T. and E.T. under different time points were given based on three times measurements. Error bars were defined as s.d. (**h**) Finite element method (FEM) simulation of the heat conduction of a single csUCNP@C nanoparticle induced by the photothermal process. Scale bar, 100 nm.

The synthetic procedure of csUCNP@C is shown in Supplementary Fig. 1. The carbon shell generated heat under 730-nm irradiation and the core of NaLuF_4_:Yb,Er provided thermal-sensitive upconversion luminescence (UCL) emission under 980-nm excitation (Fig. 1). As shown by transmission electron microscopy (TEM), the NaLuF_4_:Yb,Er nanoparticles (UCNPs, the core) were uniform in morphology with a diameter of ∼25 nm (Fig. 2b). After coating with a non-doping NaLuF_4_ layer, the shape of the formed nanoparticles was changed to rod-like and the size was increased to ∼50 nm in length and ∼40 nm in width (Fig. 2b), indicating the formation of the core-shell nanoparticles NaLuF_4_:Yb,Er@NaLuF_4_ (csUCNPs). X-ray powder diffraction (Supplementary Fig. 2c) patterns of UCNPs and csUCNPs were indexed as the hexagonal phase of NaLuF_4_. The as-prepared oleate-capped csUCNPs were transformed into the aqueous phase using an acid-based ligand removal method reported by Capobianco *et al*.^44^ To coat the shell layer of carbon, a glucose solution (0.4 mmol ml^−1^) containing hydrophilic csUCNPs (2 mg ml^−1^) was hydrothermally treated at 160 °C for 2 h. Fourier-transform infrared spectroscopy indicated that the oleate species on csUCNPs were totally removed after acid treatment (Supplementary Fig. 4a) and a carbon layer was successfully coated on csUCNPs after the hydrothermal treatment, as the stretching bands referred to as C−H and C=C bonds appeared. A new coating layer was also visible in the TEM images (Fig. 2b; Supplementary Fig. 2b) and the monodispersity of the nanoparticles kept good (Supplementary Fig. 3). In particular, Raman spectra confirmed the dominance of an ordered conjugated *π*-bond structure in the outside shell layer, as the intensity ratio of the peaks of graphitic carbon and amorphous carbon reached 4.3 (Supplementary Fig. 4c). The highly graphitic components in the carbon shell, inducing effective *π*-plasmon, accounted for the broad absorption from visible to NIR region of csUCNP@C (Fig. 2d; Supplementary Fig. 5). Thus, the colour of the nanocomposites turned from white of csUCNP to deep brown (Fig. 2d inset). After carbon coating, the average hydrodynamic diameter was increased from 56.5 to 77.0 nm (Supplementary Fig. 6b), and the weight percentage (wt%) of the organic components was enhanced from ∼0.5 to ∼3.7wt% (Supplementary Fig. 4d). These factors indicated that a carbon layer was successfully coated on the surface of csUCNPs by hydrothermal treatment. The as synthesized csUCNP@C can be easily dispersed in water and other buffers because polymerization of glucose occurred before carbonization and the hydrophilic polymer chains located at the outer layer of csUCNP@C, which make the nanoparticles very stable in aqueous phase^45^. Dynamic light scattering (DLS) data showed that the colloidal suspensions can be preserved for 2 weeks without any aggregation (Supplementary Fig. 7).

Importantly, the insertion of a non-doping NaLuF_4_ interlayer (∼7.5-nm thick) between the carbon shell and upconversion core of NaLuF_4_:Yb,Er can both enhance the UCL emission and prevent luminescent quenching by the carbon shell. When the concentration of the luminescence centre of Er^3+^ was consistent, csUCNPs emitted stronger UCL at 540 nm (increased 4.9-fold) compared with UCNPs. The control NaLuF_4_:Yb,Er@Carbon (UCNP@C) without a NaLuF_4_ protective layer displayed an 83% decrease in UCL intensity, whereas csUCNP@C with a protective layer maintained 82% of the UCL intensity (Fig. 2c). Thus, the introduction of NaLuF_4_ is necessary for fabricating the temperature-feedback PTT agent.

Photothermal properties of csUCNP@C were also evaluated by measuring the photothermal conversion efficiency under 730-nm laser irradiation (1 W cm^−2^). Detailed data are shown in Supplementary Information (Supplementary Figs 8 and 9). The final heat-generation efficiency (*η*) is 38.1 % that is higher than those widely studied PTT agents such as gold nanorods (21%) and nanoshells (13%), Cu_2−*x*_Se (22%) and Cu_9_S_5_ (25.7%)^46^,^47^. The high heat-generation efficiency indicated that the carbon shell of csUCNP@C is a kind of excellent PTT agent to realize the therapeutic temperature at even lower laser-irradiation dosage. It should be noted that the 2-in-1 design that combined temperature-sensitive upconversion luminescence with photothermal carbon shell can realize both good temperature-sensing property and photothermal effect. Although other carbon materials such as carbon dots also exhibit special luminescent property that is marked by tunable emission wavelength and broad excitation spectra^48^,^49^, their absorption spectrum usually located in ultraviolet to blue region and their photothermal effect under NIR laser irradiation has not been proved yet. Also, the temperature-sensing properties of carbon dots have not been clarified.

### Observation of the eigen temperature of csUCNP@C

In the Er^3+^-doped upconversion system, ^2^H_11/2_→^4^I_15/2_ (UCL emission centred at 525 nm) and ^2^S_3/2_ → ^4^I_15/2_ (centred at 545 nm) transitions were in close proximity to a thermal equilibrium ruled by the Boltzmann factor (equation 1)^24^:


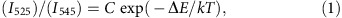


where *I*_525_ and *I*_545_ are the UCL emission of the ^2^H_11/2_ → ^4^I_15/2_ and ^4^S_3/2_ → ^4^I_15/2_ transitions, respectively; *C* is a constant determined by the degeneracy, spontaneous emission rate and photon energies of the emitting states in the host materials; Δ*E* is the energy gap separating the two excited states; *k* is the Boltzmann constant; *T* is temperature using the Kelvin scale. The changes in the UCL intensities as a function of temperature make it possible to quantitatively monitor the eigen temperature fluctuation of csUCNP@C when irradiated.

To simultaneously measure the apparent temperature and upconversion emission spectrum of the aqueous solution containing csUCNP@C, we designed and set-up a system by introducing a thermometer and upconversion emission spectroscope, as shown in Fig. 2a. First, we obtained a calibration curve to determine the relationship between UCL intensity and temperature. By heating the solution of csUCNP@C with a temperature controller (Supplementary Fig. 10), the UCL emission at 525 nm was correspondingly enhanced (Fig. 2e). The dependence of ln(*I*_525_/*I*_545_) on the inverse temperature (1/*T*), which showed a linear behaviour (Fig. 2f), was well fitted as ln(*I*_525_/*I*_545_)=1.085−0.838 × (1/*T*) (*T* given in K). Judging from the signal changes of UCL intensity changes, csUCNP@C is a good optical nanothermometer with a relatively high sensitivity of 1% signal change K^−1^ around 35–40 °C and a high temperature resolution of about 0.5 K. Although some previous works have reported other methods for intracellular thermometry with excellent temperature resolution and sensitivity^50^,^51^,^52^, those thermometry methods rely on single band emission without an internal ratiometric. Hence, the accuracy of temperature sensing will be strongly affected by other environmental factors, which can change the emission intensity such as absorption, scattering, tissue motions, autofluorescence and quenching centres. Moreover, their combination with photothermal therapy have not been exploited and the advantages of marked local temperature changes of PTT agents have not been fully recognized to improve PTT. The optical temperature sensor in csUCNP@C, NaLuF_4_:Yb,Er, has a couple of temperature-sensitive emission bands (centred at 525 and 545 nm) for ratiometric thermometry, which is insusceptible to disturbance of environmental factors. The closely combined structure of temperature sensor and photothermal component in csUCNP@C is indispensable to monitor local temperature elevation of PTT agents during photothermal process and thus give us the chance to explore a new strategy of photothermal therapy with much less normal tissue damage.

To observe the difference of eigen and apparent temperature, csUCNP@C solution under 730-nm irradiation at various time points were recorded using the thermometer (Fig. 2a) to compare the eigen temperature of csUCNP@C and the apparent temperature. As shown in Fig. 2g, the eigen temperature of csUCNP@C was much higher than the apparent temperature at each time point, with a significant difference of ∼34 K at equilibrium with a power density of 0.8 W cm^−2^ at 730-nm irradiation. Under low-power illumination at 0.3 W cm^−2^ for 8 min, the apparent temperature of the solution was 36.6 °C, whereas the eigen temperature of csUCNP@C soared to 65.5 °C by determining the ratio of UCL emission at 525 to 545 nm. Even when irradiated for 2 min, the eigen temperature of csUCNP@C reached 59.7 °C, whereas the apparent temperature of the solution was 31.6 °C. To the best of our knowledge, this is the first time such a significant difference between eigen temperature of the PTT agent and apparent temperature of its surrounding system has been reported. It should be noted that NaLuF_4_:Yb,Er as temperature sensor has a temperature resolution of 0.5 °C and sensitivity of 1% signal change per degree, which can be perfectly qualified for differentiating the remarkable temperature elevation (for example, eigen temperature elevation is 30.0 °C with 730-nm irradiation at 0.3 W cm^−2^ for 2 min) of PTT agents in microscopic state.

To shed light on the temperature distribution at the microscopic level, we used a single-particle model to simulate the possible temperature distribution around the nanoparticles (heating centre) in solution. As shown in Fig. 2h, the temperature of the nanoparticles was kept at 80 °C (in accordance with the highest eigen temperature in our experiment). Water was chosen as the ambient condition for the nanoparticles and the external boundary was kept at 35 °C. The model simulated a steady state with heat conduction mode (see Methods). The simulation showed that the temperature of water declined with increasing distance from the nanoparticles, below the lethal temperature to cancer cells (39 °C) at ∼360 nm in diameter (Fig. 2h). Therefore, it is reasoned that under 730-nm laser irradiation, csUCNP@C in the aqueous phase become localized hot spots whose eigen temperature is much higher than the ambient temperature, and thus their effective range for killing cancer cells by PTT is in the nanoscale. Therefore, it was theoretically proved that PTT can be performed at low apparent temperature and high spatial accuracy.

### Facile and high-accuracy PTT *in vitro*

**Figure 3:**
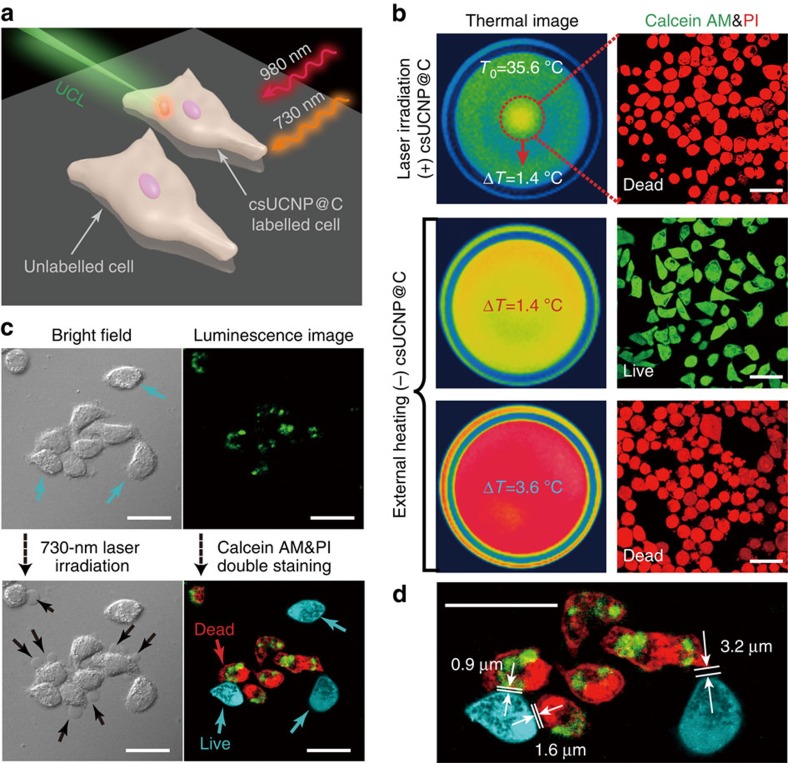
csUCNP@C for high-accuracy PTT at cell level. (**a**) Schematic diagram of PTT in cells. (**b**) Thermal images and Calcein AM and PI double-stained images of HeLa cells treated with photothermal ablation or external heating. Non-labelled cells were irradiated by 730-nm laser (0.3 W cm^−2^) and the final apparent temperature elevation in the laser spot (*T*_0_=35.6 °C) was set as a benchmark. Δ*T* was the difference between apparent temperature and *T*_0_. In external heating, cells were alive when Δ*T*=1.4 °C and dead when Δ*T*=3.6 °C. With 730-nm laser irradiation, csUCNP@C labelled cells were dead when Δ*T*=1.4 °C indicating that the eigen temperature of csUCNP@C had reached to a lethal temperature to the cells even though the apparent temperature was still safe. Scale bar, 50 μm. (**c**) Photothermal therapy of HeLa cells under 730-nm laser irradiation at 0.3 W cm^−2^ for 5 min. Cells labelled with csUCNP@C showed a strong UCL signal in the cytoplasm (green). The signal is collected in the wavelength region of 520–550 nm. After 730-nm irradiation, dead cells showed conspicuous cytoplasm leakage which labelled with black arrows. Calcein AM (cyan) and PI (red) double-staining showed that only the cells labelled with csUCNP@C were dead. Scale bar, 30 μm. (**d**) Amplified image of the luminescent cell images in **c**. The distance between the adjacent live and dead cells was measured. The minimum distance was ∼0.9 μm. Scale bar, 30 μm.

To confirm that PTT is effective at low apparent temperature, experiments at the macroscopic level were carried out. First, non-labelled HeLa cells were irradiated by a 730-nm laser (0.3 W cm^−2^) to figure out the laser-induced heating effect and the final apparent temperature elevation in the laser spot, *T*_0_ (35.6 °C, Supplementary Fig. 13b), was set as a benchmark. Δ*T* shown in Fig. 3b was the temperature increment subtracting T_0_ after photothermal process or external heating. Calcein acetoxymethyl ester (Calcein AM) and propidium iodide (PI) double staining were used to confirm the state (live or dead) of cells. When csUCNP@C-incubated HeLa cells were irradiated under 0.3 W cm^−2^ for less than 3 min, Calcein AM/PI staining indicated that csUCNP@C-incubated cells were dead when Δ*T* was 1.4 °C. Non-labelled cells with external heating holder indicated that cells were alive when Δ*T* was 1.4 °C and were dead only when Δ*T* reached 3.6 °C. These results suggest that the photothermal effect of csUCNP@C can effectively kill the cells without heating the surrounding solution to a high apparent temperature.

As proof of concept experiments to investigate the accuracy of PTT, we mixed csUCNP@C-labelled HeLa cells with non-labelled cells together and let them adhere to the culture dish. In our designed PTT system (Fig. 3a), two lasers, one at 980 nm and the other at 730 nm, were used to generate upconversion emission and the photothermal effect of csUCNP@C, respectively. Before 730-nm irradiation, csUCNP@C-treated cells (incubating dosage, 200 μg ml^−1^), which have a relatively wide distribution of csUCNP@C in cytoplasm with a uptake of 4.4 pg csUCNP@C per cell, could be stained with Calcein AM (Supplementary Fig. 11a–d) showing they were all alive. Methyl thiazolyl tetrazolium (MTT) assays also confirmed that csUCNP@C have no toxic effect on cells under this incubating dosage (Supplementary Fig. 11e,f). A low-power density (0.3 W cm^−2^) of 730-nm laser was adopted for photothermal cell ablation. Determined by UCL intensity changes on the csUCNP@C-labelled HeLa cells (Supplementary Fig. 14), a huge eigen temperature elevation was observed after 730-nm laser irradiation from 37 °C to nearly 60 °C (Supplementary Fig. 15). Such a high eigen temperature reflects enormous localized heat which is very critical in killing the cancer cells. As shown in Fig. 3c, only the cells with upconversion emission displayed red fluorescence from PI (*λ*_ex_=633 nm), whereas the others without upconversion signals showed the fluorescence signal from Calcein AM (cyan, *λ*_ex_=488 nm). The results indicated that, after 730-nm irradiation, the csUCNP@C-labelled cells were dead and the free cells were still alive. Bright-field image of the csUCNP@C-labelled cells showed membrane damage and leakage of the cytoplasm. Moreover, laser irradiation of csUCNP@C-labelled HeLa cells and non-labelled C2C12 cells indicated that photothermal effect can only occurred in the cancer cells without damaging normal cells (Supplementary Fig. 12). MTT assays also confirmed these results by a remarkable decrease in cell viability after 730-nm irradiation (Supplementary Fig. 10f). By further decreasing the power density of the 730-nm laser to 0.2 W cm^−2^, early stage cell apoptosis was observed only in csUCNP@C-labelled cells (Supplementary Fig. 13a), using the Annexin V-FITC/PI double staining method. On the basis of the cell selective ablation experiment (Fig. 3b,c; Supplementary Fig. 15), we can conclude that high temperature of a limited space is enough to kill cells, while the overall temperature changes little. In other words, with the microscopic temperature monitoring technology, there is no longer a need to use overall temperature to monitor PTT. The usage of overall temperature of cells for PTT, which only involves the average value of high-temperature region containing csUCNP@C and low-temperature region without csUCNP@C, will neglect the significance of localized high temperature in killing the cancer cells and obviously restrict the therapeutic accuracy. It is worth noting that the distance between PTT-affected cells and unaffected cells in the confocal image (Fig. 3c,d) was at the micrometre level and the minimum distance was only 0.9 μm, that is, the photothermal ablation under a low apparent temperature was proved to have very high spatial resolution at the microscopic level.

### Facile and high-accuracy PTT *in vivo*

**Figure 4:**
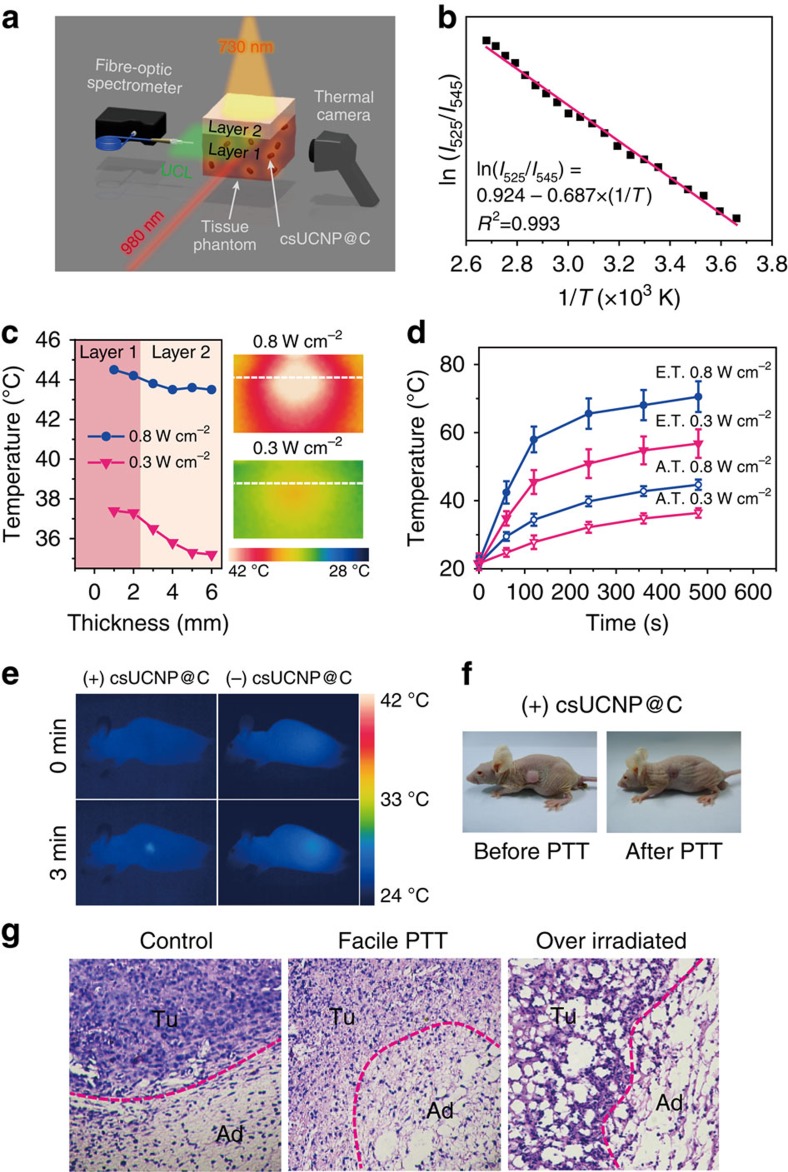
csUCNP@C for high-accuracy PTT *in vivo*. (**a**) Schematic diagram of the feasibility of PTT *in vivo* using a tissue phantom. (**b**) A plot of ln(*I*_525_/*I*_545_) versus 1/*T* to calibrate the thermometric scale for csUCNP@C in the tissue phantom. (**c**) Apparent temperature versus the thickness of the tissue phantom (from layer 1 to layer 2) under 730-nm irradiation at two different power densities (left panel). Thermal images of longitudinal sections of the phantom within two irradiation power densities to show the heat conduction process. The white dashed line separates the simulated ‘tumour' and ‘normal tissue' (right panel). (**d**) Elevation of apparent temperature (A.T.) and eigen temperature (E.T.) of csUCNP@C in the tissue phantom under irradiation with 730-nm laser at 0.8 and 0.3 W cm^−2^. Average values of A.T. and E.T. under different time points were given based on three times measurement. Error bars were defined as s.d. (**e**) Thermal images of nude mice with (left panel) and without (right panel) csUCNP@C-labelled HeLa cell tumours under 730-nm irradiation (0.3 W cm^−2^). (**f**) Representative photos of nude mice transplanted with csUCNP@C-labelled HeLa cells under 730-nm irradiation (0.3 W cm^−2^). (**g**) H&E histologic section of the border of tumour and normal fat tissue. The tumour region (Tu) and the adipocytes (Ad) in normal fat tissue of the mice without 730-nm irradiation (left, control) is compact and the tumour cells are stretched. Following photothermal treatment (middle, Facile PTT), the tumour region became loose and fragile and the tumour cells are atrophic. The adipocytes (Ad) in normal fat tissue are intact with minimal damage. However, following high-power irradiation with the 730-nm laser (0.8 W cm^−2^), both Tu and Ad suffered extreme damage (right, Over irradiated).

To further confirm the feasibility of photothermal therapy at low apparent temperature and investigate the heat-conduction process in the living body, it is crucial to know about the eigen temperature fluctuations of csUCNP@C under laser irradiation in biological tissue. As it is difficult to obtain the temperature calibration curve and to investigate the heat conduction process in living animals due to some limiting factors in instruments, tissue phantoms were used to simulate the temperature elevation of csUCNP@C in biological tissue (Fig. 4a). The tissue phantom is consisted of gelatin and a certain amount of haemoglobin and intralipid to simulate the absorption and scattering properties of real tissue (see Supplementary Methods for the synthesis of tissue phantom). The temperature calibration curve (from 0 to 100 °C) obtained in a single-layer phantom containing csUCNP@C showed a linear behaviour [ln(*I*_525_/*I*_545_)=0.924−0.687 × (1/*T*)] (*T* given in K, Fig. 4b), which was similar to that detected in solution (Fig. 2f). A double-layer phantom where the lower layer of the phantom containing csUCNP@C (Layer 1, ∼10-mm thick) simulating the tumour area for therapy and the upper layer without csUCNP@C (Layer 2, ∼4-mm thick) simulating normal tissue (Fig. 4a) was used to investigate the heat conduction in facile and excessive photothermal process. The photothermal effect of csUCNP@C in the phantom tissues was triggered by 730-nm irradiation at 0.3 or 0.8 W cm^−2^. The apparent temperatures of layer 1 (‘tumour') and layer 2 (‘normal tissue') were recorded using a thermal camera, and the eigen temperature of csUCNP@C in layer 1 was calculated by the upconversion emission spectrum (Fig. 4a). Following 0.8 W cm^−2^ irradiation for 8 min, layer 1 was heated to 44.5 °C (apparent temperature) and layer 2 also underwent an obvious temperature elevation to 43.3 °C due to massive heat conduction, that is, the overheating photothermal effect. However, following mild laser irradiation at 0.3 W cm^−2^, the apparent temperature of layer 1 was moderate at 37.3 °C and layer 2 only showed slight heat conduction (35.2 °C), as shown in Fig. 4c. Meanwhile, the eigen temperature of csUCNP@C within the phantom obtained by the UCL spectrum was 56.7 °C, which is sufficient to kill cancer cells (Fig. 4d). Therefore, it can be safely concluded that csUCNP@C as a PTT agent can take effect under mild apparent temperature elevation, while excessive laser power aggravates injury of normal tissue. It should be noted that in previous publications on PTT, an overheating effect existed during the therapy process due to the absence of a temperature-feedback unit in the photothermal agent.

The above-mentioned results encouraged us to further assess the therapeutic effect of csUCNP@C in small living animals. The csUCNP@C-incubated (4.4 pg per cell) HeLa cells (1 × 10^7^ cells) were subcutaneously transplanted into nude mice for tumour growth. Fourteen days after transplantation, a tumour with a diameter of ∼0.6 cm was observed and displayed strong UCL signals collected by a 720-nm short pass filter (Fig. 4f; Supplementary Fig. 19a). The apparent temperature in the tumour area was recorded by an infrared thermal imaging device. The eigen temperature in the tumour area was calculated by upconversion luminescence of csUCNP@C. On the basis of the therapeutic data and the heat conduction behaviour under two sets of 730-nm laser power density (0.3 and 0.8 W cm^−2^) in Fig. 4c, we have summarized and proposed a model of temperature-feedback photothermal treatment system and make a demonstration of this feedback treatment with the strategies given in this work (Supplementary Fig. 18). In this model, controller (in this work, controller is experimenter) will make decision on the treatment strategy. Strategy box stores a series of photothermal therapy strategies for selection (in this work, strategies are 1# and 2# with 730-nm laser at 0.3 and 0.8 W cm^−2^, respectively.). csUCNP@C receives the strategy output and is served as photothermal agent and eigen temperature reporter for feedback signal input. Eigen temperature of csUCNP@C with different power density of 730-nm laser irradiation reported by UCL spectra (Supplementary Fig. 17) showed that both sets of power density can result in an enough high eigen temperature to ablate cancer cells (61.5 °C with 0.3 W cm^−2^ and 73.1 °C with 0.8 W cm^−2^). The temperature elevation through heat conduction in layer 2 (‘normal tissue') is confined within 2 °C under 0.3 W cm^−2^ irradiation. Considering that the body temperature is 37 °C and 2 °C elevation will not exceed 40 °C that cause protein denaturation^53^, treatment strategy 1# with a power density of 730-nm laser at 0.3 W cm^−2^ was chosen for therapy. Tumour-bearing mice with csUCNP@C-labelled were exposed to a 730-nm laser (0.3 W cm^−2^) for 3 min and the difference between final apparent temperature (at 3 min) and the initial temperature (at 0 min) was controlled at ∼1.5 K (Fig. 4e). The tumour-bearing mice without csUCNP@C treatment (control) under 0.3 W  cm^−2^ 730-nm laser irradiation showed a slight increase in apparent temperature (Fig. 4e). The tumour in each mouse in the treatment group was exposed to the 730-nm laser every day. Five days later, the tumours in the treatment group shrank and were finally eliminated without any regrowth (Supplementary Figs 19b and 21; Supplementary Table 1). In contrast, neither csUCNP@C-treatment nor laser irradiation affected tumour growth and the tumour size increased rapidly. Reference groups (including untreated mice, mice exposed to the 730-nm laser only, and csUCNP@C-treated mice without 730-nm irradiation) showed an average lifespan for tumour-bearing mice of ∼22 days, while the mice in the PTT experimental group survived for over 40 days without mortality (Supplementary Fig. 19b,c). Hematoxylin and eosin (H&E) histopathological analysis indicated that malignant cells at the tumour site (Tu) in the facile PTT treatment group (730-nm laser, 0.3 W cm^−2^) showed obvious shrinkage and fragmented nuclei (Fig. 4g), whereas the control group showed no conspicuous necrosis (Fig. 4g). Furthermore, the adjacent subcutaneous adipocytes (Ad) showed intact morphology in the control group and in the facile PTT treatment group (0.3 W cm^−2^), while both Tu and Ad sites in the 730-nm over-irradiated group (0.8 W cm^−2^) that had a high apparent temperature (Supplementary Fig. 16) were severely damaged (Fig. 4g). Hence, by using csUCNP@C as a photothermal agent, PTT at a mild apparent temperature is successfully achieved in living animals without damaging normal tissue.

Furthermore, using folic-acid-modified csUCNP@C (FA-csUCNP@C) as the photothermal agent, targeting PTT of the HeLa tumour-bearing balb/c mice under mild apparent temperature was investigated. After intravenous injection of FA-csUCNP@C (2 mg ml^−1^, 200 μl) for 2 h, strong UCL signals were detected in tumour region (Supplementary Fig. 22a). *Ex vivo* UCL imaging indicated the biodistribution of csUCNP@C in other organs (Heart, Liver, Spleen, Lung, Kidney and Tumour) (Supplementary Fig. 22b). Histological and serum biochemistry assays suggested no evident toxic effects *in vivo* within one week of FA-csUCNP@C administration when compared with the untreated group (Supplementary Fig. 20; Supplementary Methods for detailed experimental procedures). After 730-nm irradiation (0.3 W cm^−2^) for 3 min three times per day for 6 days, the tumours shrank and were finally eliminated (Supplementary Figs 22c and 23; Supplementary Table 2), and the PTT-treated mice survived for over 2 months without mortality (Supplementary Fig. 22d). Thus, we have successfully proved that FA-csUCNP@C can be used as a theranostic agent *in vivo*.

## Discussion

We demonstrated carbon-coated core-shell upconversion nanocomposite NaLuF_4_:Yb,Er@NaLuF_4_@Carbon (csUCNP@C) for monitoring of microscopic temperature in photothermal process. Under laser irradiation at 730 nm, the carbon shell serves as an excellent photothermal agent for cancer therapy and simultaneously heats up the nanocomposite. By analysing upconversion luminescence emitted from the NaLuF_4_:Yb,Er core of csUCNP@C during the photothermal process, microscopic temperature of photothermal agent was detected and found to be much greater than the temperature at macroscopic level. By utilizing this phenomenon, we selectively ablated csUCNP@C-labelled cancer cells under mild apparent temperature without harming adjacent non-labelled cells. The minimum separation between ablated cell and ambient preserved cell is 0.9 μm. High spatial resolution photothermal ablation *in vivo* of tumour with minimal damage to normal tissues was also realized at low apparent temperature. In stark contrast to the existing principle of PTT focusing on elevating the apparent temperature to overheating level, which can cause severe adverse effects in normal tissues near the tumour area, our approach relying on csUCNP@C as a temperature-feedback photothermal agent indicated that an effective photothermal treatment with high accuracy can be realized under moderate conditions. This point proves that the strategy to ensure enough heating of the photothermal agent to ablate the labelled cancer cell and to simultaneously circumvent heat conduction to non-labelled normal tissues is possible. The indispensable advantage of this work is to use a microscopic temperature-feedback system to point out an optimized irradiation dose for facile photothermal therapy, which is different from the common understanding and cannot be achieved by previous macroscopic temperature measuring method. If automatic controlling device with automatic spectrum analysing and laser power controlling abilities is integrated, then the strategies of temperature-feedback photothermal therapy will be more diversified and can be conducted more conveniently. It is reasonable to presume that PTT possessing high therapeutic accuracy and mild treatment conditions can do more sophisticated operations such as precise lymphadenectomy, embolization of tumour microvessels and low-injury intervention treatment around vital organs. Our work presented a powerful tool to give an insight into the photothermal process at microscopic level. By utilizing the merits of the presented temperature-feedback upconversion nanocomposite, the mode and concept of PTT will be changed profoundly.

## Methods

### Synthesis of oleate-coating NaLuF_4_:Yb,Er/Tm nanoparticles

Spherical-like oleate-coating NaLuF_4_:Yb,Er nanoparticles (OA-UCNPs) were synthesized via a modified solvothermal method^54^. In a typical procedure, 1 mmol lanthanide chloride (78% mol Lu, 20% mol Yb and 2% mol Er) were mixed with 6 ml oleic acid (OA) and 17 ml 1-octadecene in a 100-ml three-necked flask. The resulting mixture was degassed at 90 °C for 20 min, and then heated to 160 °C for 1 h to form a transparent solution. After that, the solution was cooled to room temperature. Then 2.5 mmol NaOH and 4 mmol NH_4_F dissolved in 5 ml methanol were added and the mixture was degassed for another 30 min at 90 °C. Thereafter, the solution was heated to 300 °C as quickly as possible and the temperature was maintained for 1 h under an argon atmosphere. When the reaction was complete, an excess amount of ethanol was poured into the solution at room temperature. Nanoparticles were collected by centrifugation and washed three times with ethanol/cyclohexane (1:1 v/v). The as-obtained nanoparticles OA-UCNPs were dispersed in 5 ml cyclohexane for the following synthesis.

### Synthesis of NaLuF_4_:Yb,Er@NaLuF_4_ nanoparticles

Rod-like oleate-coating NaLuF_4_:Yb,Er@NaLuF_4_ nanoparticles (OA-csUCNPs) were prepared by epitaxial growth on OA-UCNPs via a similar solvothermal method. One millimole of LuCl_3_ was mixed with 12 ml oleic acid and 15 ml 1-octadecene in a 100-ml three-necked flask. Analogous to the procedure in synthesizing OA-UCNPs, the resulting mixture was degassed at 90 °C for 20 min, and then heated to 160 °C for 1 h to form a transparent solution and then cooled to room temperature. After that, 5 ml cyclohexane solution containing OA-UCNPs was added into the system dropwise. The system was kept at 80 °C for 30 min to evaporate cyclohexane. Then 2.5 mmol NaOH and 4 mmol NH_4_F dissolved in 5 ml methanol were added into the mixture, degassed at 90 °C for 30 min, and finally maintained at 300 °C for 1 h. OA-csUCNPs were collected by centrifugation, washed three times with ethanol/cyclohexane (1:1, v/v), subsequently washed with acetone and isolated by centrifugation. The products were dried and stored for further use.

### Synthesis of carbon-coated csUCNPs

The OA-csUCNPs underwent a ligand-exchange process to make them water-soluble according to a reported method^44^. Briefly, OA-csUCNPs were dispersed in 10 ml aqueous solution (pH=4) by adding 0.1 mol l^−1^ HCl and stirring for 2 h. Diethyl ether was used to extract oleic acid yielded from the protonated oleate ligand. After extraction was carried out three times, the products in the water layer were collected by centrifugation and washed three times with deionized water. The resulting ligand-free csUCNPs were easily dispersed in water. Then a certain amount of ligand-free csUCNPs were added into 0.4 M glucose aqueous solution and were redispersed by sonication. The concentration of nanoparticles was kept at 2 mg ml^−1^. The solution was transferred to a 50-ml autoclave, sealed and hydrothermally treated at 160 °C for 2 h. Finally, the system was allowed to cool to room temperature and carbon-coated csUCNPs (csUCNP@C) were collected by centrifugation and washed three times with deionized water.

### Synthesis of folic acid-conjugated csUCNP@C

To synthesize FA functionalized csUCNP@C, 10 mg FA was mixed with 10 mg 4-dimethylaminopyridine and 20 mg csUCNP@C in 2 ml anhydrous dimethylformamide under a N_2_ atmosphere. sixteen milligrams of *N*,*N*′-dicyclohexylcarbodiimide and 20 μl triethylamine was then added into the mixture and stirred for 24 h. An excess of diethyl ether was added to precipitate FA-conjugated csUCNP@C (FA-csUCNP@C). The as obtained FA-csUCNP@C were washed with methanol once and washed with ethanol twice and finally redispersed in deionized water.

### Simulation of temperature distribution in one-nanoparticle system

Here we used a finite element method to simulate the heat conduction process of a single nanoparticle in the aqueous phase. An ellipse with a long axis of 50 nm and short axis of 40 nm was set as the nanoparticle. A circle with a diameter of 1 μm was set as the water surroundings. The temperature of the nanoparticle was set at 80 °C and the external boundary of water was set at 35 °C. The model only considered the heat-transfer process in the static condition. The temperature distribution presented is the final equilibrium state. The initial temperature was set at 35 °C.

### Calculation of the photothermal conversion efficiency

According to the method described in the literature, the total energy conservation for the system can be expressed by equation 2.


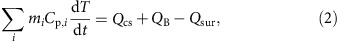


where *m* and *C*_p_ are the mass and heat capacity of water, respectively, *T* is the solution temperature, *Q*_cs_ is the energy induced by carbon shell of csUCNP@C, *Q*_B_ is the baseline energy induced by the sample cell, and *Q*_sur_ is heat conduction away from the surface by air.

*Q*_cs_ is caused by the *π*-plasmon of the carbon shell under irradiation of 730-nm laser:


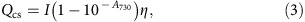


where *I* is the laser power, *η* is the conversion efficiency from incident laser energy to thermal energy, and *A*_730_ is the absorbance of carbon shell of csUCNP@C at wavelength of 730 nm. On the other hand, *Q*_*B*_, expressing heat dissipated from light absorbed by the sample cell, was measured independently to be 28.4 mW using a quartz cuvette containing pure water without csUCNP@C. Moreover, *Q*_*sur*_ is in proportion to temperature for the outgoing thermal energy, as given by equation 4:


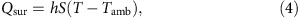


where *h* is heat transfer coefficient, *S* is the surface area of the container, and *T*_amb_ is ambient temperature of the surroundings.

According to equation 4, when the system temperature will reach a maximum, the heat input is equal to heat output:


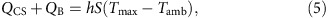


where *T*_max_ is the equilibrium temperature. The 730-nm laser heat-conversion efficiency (*η*) can be determined by substituting equation 3 for *Q*_cs_ into equation 5 and rearranging to get


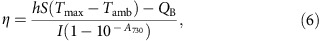


where *Q*_B_ was measured independently to be 28.4 mW, the (*T*_max_−*T*_amb_) was 22.3 °C according to Supplementary Fig. 8a, *I* is 1 W cm^−2^, *A*_730_ is the absorbance (1.254) of csUCNP@C at 730 nm (Supplementary Fig. 9). Here *hS* is calculated by introducing *θ*, is defined as the expression below:


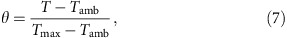


and a sample system time constant *τ*_*s*_


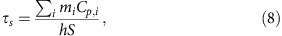


which is substituted into equation 5 and rearranged to yield


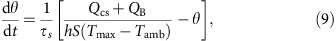


At the cooling stage of the aqueous dispersion of the csUCNP@C, the light source was shut off, the *Q*_cs_+*Q*_B_=0, reducing the equation 10:


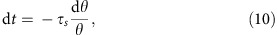


and integrating, giving the expression:


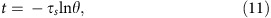


Therefore, time constant for heat transfer from the system is determined to be *τ*_*s*_=120.5 s by applying the linear time data from the cooling period (after 400 s) versus negative natural logarithm of *θ* (Supplementary Fig. 8b). In addition, the *m* is 0.5 g and the *C* is 4.2 J g^−1^. Thus, according to equation 11, the *hS* is deduced to be 17.4 mW °C^−1^. Substituting 17.4 mW °C^−1^ into the *hS* into equation 6, the 730-nm laser heat conversion efficiency (*η*) of csUCNP@C can be calculated to be 38.1%.

### Eigen temperature measurement of csUCNP@C in solution

To obtain temperature-upconversion luminescence calibration curve in solution (Apparatus diagram is shown in Supplementary Fig. 10.), a quartz cuvette containing csUCNP@C aqueous dispersion (2 ml, 0.5 mg ml^−1^) was placed in Edinburgh FLS-920 fluorescence spectrometer with an external temperature controller. Aqueous solution was heated to different temperature from 5 to 100 °C and the corresponding UCL from 500 to 580 nm was recorded with excitation of a continuous wave (CW) 980-nm laser (50 mW cm^−2^). The evolutions of the ratio of UCL emission peaks centred at 525 and 545 nm as function of temperature are used as calibration curve for eigen temperature monitoring. To evaluate the photothermal process of csUCNP@C (Apparatus diagram is shown in Fig. 2a.), a quartz cuvette containing an aqueous dispersion (2 ml) of csUCNP@C (0.5 mg ml^−1^) was irradiated with an optical fibre coupled 730-nm diode-laser (Weining Technology Development Co., Ltd. China) for 8 min at a laser power of 0.3 and 0.8 W cm^−2^. A CW 980-nm laser was used to generate the upconversion luminescence. UCL spectra, from 500 to 580 nm, were collected by Edinburgh FLS-920 fluorescence spectrometer at different time intervals (0, 40, 120, 240, 360 and 480 s). The eigen temperature of csUCNP@C was determined from the ratio of the luminescence peaks which centred at 525 nm and 545 nm. Apparent temperature changes of the solution were recorded by a thermocouple thermometer.

### Cell culture and confocal UCL imaging *in vitro*

HeLa cells and C2C12 myoblasts were provided by the Institute of Biochemistry and Cell Biology, SIBS, CAS (China). The cell lines used in this work (HeLa cells and C2C12 myoblasts) do not appear in the list of mis-identified cell lines made by International Cell Line Authentication Committee (ICLAC). Cells were grown in RPMI 1640 (Roswell Park Memorial Institute medium) supplemented with 10% fetal bovine serum at 37 °C and 5% CO_2_. Cells (5 × 10^8^ l^−1^) were plated on 14-mm glass coverslips under 100% humidity and allowed to adhere for 24 h. After washing with PBS, the cells were incubated in a serum-free medium containing 200 μg ml^−1^ csUCNP@C at 37 °C for 2 h under 5% CO_2_, and then washed with PBS three times to get rid of excess nanoparticles. Confocal UCL imaging was performed on our designed laser scanning UCL microscope with an Olympus FV1000 scanning unit. The set-ups of the confocal UCL microscopy and upconversion luminescence *in vivo* imaging system are detailed in ref. 55, 56, respectively. The cells were excited by a CW laser operating at 980 nm (Connet Fiber Optics, China) with the focused power of ∼19 mW. A 60 × oil-immersion objective lens was used and luminescence signals were detected in the wavelength region of 500–580 nm. To qualitatively assess the photothermal effect *in vitro*, cells were irradiated with a 730-nm laser at a power density of 0.3 W cm^−2^ for 5 min, and then stained with PI (propidium iodide) and calcein AM. PI signals were collected at 600-680 nm excited with a CW 543 nm laser. Calcein AM signals were collected at 500–580 nm excited with a CW 488-nm laser. To assess the apoptosis promoting effect of csUCNP@C *in vitro*, cells were irradiated with a 730-nm laser at a low-power density of 0.2 W cm^−2^ for 5 min, and then stained with PI and Annexin V-FITC. Annexin V-FITC signals were collected at 500–550 nm excited with a CW 488-nm laser.

### Temperature mapping of csUCNP@C labelled cells

For eigen temperature monitoring in cell, confocal UCL imaging was performed on our designed laser scanning UCL microscope with an Olympus FV1000 scanning unit. The HeLa cells were incubated in a serum-free medium containing 200 μg ml^−1^ csUCNP@C at 37 °C for 2 h under 5% CO_2_, and then washed with PBS three times to get rid of excess nanoparticles. Then csUCNP@C labelled HeLa cells were excited by a CW laser operating at 980 nm (Connet Fiber Optics, China) with the focused power of ∼19 mW. A 60 × oil-immersion objective lens was used and luminescence signals were detected in the wavelength region of 540–570 nm (*I*_545_) and 515–535 nm (*I*_525_), respectively. Eigen temperature mapping of csUCNP@C labelled cells before and after 730-nm irradiation (0.3 W cm^−2^ for 5 min) was achieved by determining the ratio of *I*_545_ and *I*_525_ in confocal images based on calibration formula ((*I*_545_)/(*I*_525_)=*C* exp (−Δ*E*/*kT*)).

### *In vitro* photothermal cytotoxicity of csUCNP@C

*In vitro* quantitative photothermal cytotoxicity of csUCNP@C was measured by performing MTT assays on HeLa cells. Cells were seeded into a 96-well cell culture plate at 5 × 10^4^ per well, under 100% humidity, and were cultured at 37 °C and 5% CO_2_ for 24 h; different concentrations of csUCNP@C (0, 50, 100, 150, 200, 300 and 400 μg ml^−1^, diluted in RPMI 1640) were then added to the wells. The cells were subsequently incubated for 3 h at 37 °C under 5% CO_2_. Thereafter, the cells were exposed to an NIR laser (730 nm, 0.3 W cm^−2^) for 0 and 5 min, respectively, and then incubated for another 24 h. After that, MTT (5 μl; 5 mg ml^−1^) was added to each well and the plate was incubated for an additional 4 h at 37 °C under 5% CO_2_. Following the addition of 10% SDS (50 μl per well), the assay plate was allowed to stand at room temperature for 12 h. The optical density OD_570_ value (*Abs.*) of each well, with background subtraction at 690 nm, was measured by means of a Tecan Infinite M200 monochromator-based multifunction microplate reader. The following formula was used to calculate the inhibition of cell growth:


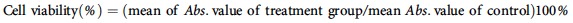


### Temperature monitoring in tissue phantom

Apparatus configuration and the measurement of UCL-temperature calibration curve are similar to that in aqueous solution, but the aqueous solution of csUCNP@C was replaced by tissue phantom (see Supplementary Methods for the synthesis of tissue phantom). To evaluate the photothermal process of csUCNP@C in tissue phantom, a double-layer phantom where the lower layer of the phantom containing csUCNP@C (Layer 1, ∼10-mm thick) simulating the tumour area for therapy and the upper layer without csUCNP@C (Layer 2, ∼4-mm thick) simulating normal tissue was prepared. Schematic diagram of temperature monitoring in tissue phantom is shown in Fig. 4a. Tissue phantom was irradiated with an optical fibre-coupled 730-nm diode-laser (Weining Technology Development Co., Ltd. China) for 8 min at a laser power of 0.3 and 0.8 W cm^−2^. A CW 980-nm laser was used to generate the UCL. UCL spectra, from 500 to 580 nm, were collected by fibre-optic spectrometer (PG2000 Pro, Ideaoptics, China) at different time intervals (0, 40, 120, 240, 360 and 480 s) for the calculation of eigen temperature. Thermal camera (FLIR E40) was used to record the apparent temperature. Investigation of heat conduction from layer 1 to layer 2 was also carried out by reading the temperature value in every other millimetre around the borderline of layer 1 and layer 2 from the thermal images.

### Tumour xenografts

Animal procedures were in agreement with the guidelines of the Institutional Animal Care and Use Committee, School of Pharmacy, Fudan University. For *in vivo* photothermal therapy with csUCNP@C pre-labelled tumour, HeLa cells were incubated in a serum-free medium containing 200 μg ml^−1^ csUCNP@C at 37 °C for 2 h under 5% CO_2_, and then washed with PBS three times to get rid of excess nanoparticles. After that, HeLa cells were collected by incubation with 0.05% trypsin-EDTA. Cells were collected by centrifugation and resuspended in sterile PBS. Cells (10^7^ cells per site) were subcutaneously implanted into 4-week-old male athymic nude mice. Photothermal therapy was performed when the tumours reached an average diameter of 0.6 cm. For *in vivo* photothermal therapy, HeLa cells were collected by incubation with 0.05% trypsin-EDTA. Cells were collected by centrifugation and resuspended in sterile PBS. Cells (10^8^ cells per site) were subcutaneously implanted into 4-week-old female athymic Balb/c mice. Photothermal therapy was performed when the tumours reached an average diameter of 0.6 cm.

### UCL bioimaging *in vivo*

UCL imaging *in vivo* was performed with an *in vivo* imaging system designed by our group, using two external 0∼5 W adjustable CW 980-nm lasers (Connet Fiber Optics Co., China) as the excited source and an Andor DU897 EMCCD as the signal collector. Excitation was provided by the CW laser at 980 nm and UCL signals were collected using a 720-nm short-pass filter. In the case of csUCNP@C-labelled HeLa cells transplantation, UCL imaging was performed when the tumour reached an average diameter of 0.6 cm. In the case of *in vivo* targeting imaging, FA-csUCNP@C were intravenously injected into HeLa cell tumour-bearing athymic Balb/c mice. Whole-body imaging of the nude mice was performed 1 h after the injection.

### Eigen temperature monitoring *in vivo*

Eigen temperature monitoring *in vivo* was conducted in csUCNP@C labelled HeLa tumour-bearing nude mice. 0–5 W adjustable CW 980-nm lasers was chosen as the excitation source of UCL and an optical fibre-coupled 730-nm diode-laser was used for photothermal excitation. Fibre-optic spectrometer was used to collect the UCL signals and a 720-nm short-pass filter was installed in front of the probe of the spectrometer. Under 730-nm laser irradiation at 0, 0.3 and 0.8 W cm^−2^ for 3 min, upconversion emission spectra were collected by fibre-optic spectrometer and the eigen temperature under different laser-power density were calculated by the luminescence spectra with the temperature calibration curve got from the tissue phantom.

### Photothermal therapy *in vivo*

An optical fibre-coupled 730-nm diode-laser (Weining Technology Development Co., Ltd. China) was used to irradiate tumours during the experiments. For photothermal treatment, the 730-nm laser beam with a diameter of ∼10 mm was focused on the tumour area at the power density of 0.3 W cm^−2^ for 3 min. Infrared thermal images were taken by an FLIR E40 thermal imaging camera. Tumour sizes of treatment group (csUCNP@C-labelled tumour with 730-nm irradiation in csUCNP@C-labelled HeLa cells transplantation and FA-csUCNP@C targeted tumour with 730-nm irradiation in targeting photothermal therapy *in vivo*) and reference groups (untreated mice, mice exposed to the 730-nm laser only, and csUCNP@C-treated mice without 730-nm irradiation) were measured every day after treatment. Each group contained five mice for relatively rational evaluation. The tumour sizes were measured using a caliper and calculated as volume=(tumour length) × (tumour width)^2^/2. Relative tumour volumes were normalized and were calculated as *V*/*V*_0_ (*V*_0_ is the tumour volume when the treatment was initiated). According to the guidelines of Institutional Animal Care and Use Committee, School of Pharmacy, Fudan University, the maximum permitted tumour size is 20 mm in an average diameter for mice. The tumours' size in this work is confined within this criterion.

## Additional information

**How to cite this article:** Zhu, X. *et al*. Temperature-feedback upconversion nanocomposite for accurate photothermal therapy at facile temperature. *Nat. Commun.* 7:10437 doi: 10.1038/ncomms10437 (2016).

## Supplementary Material

Supplementary Information Supplementary Figures 1-23, Supplementary Tables 1-2, Supplementary Methods and Supplementary References.
