# Supplementary material for: Temperature-feedback upconversion nanocomposite for accurate photothermal therapy at facile temperature
Source: Nat Commun. 2016 Feb 4;7:10437. doi: 10.1038/ncomms10437 (PMC4742858; doi:10.1038/ncomms10437)
Supplement: Supplementary Information — Supplementary Figures 1-23, Supplementary Tables 1-2, Supplementary Methods and Supplementary References. [file ncomms10437-s1.pdf]

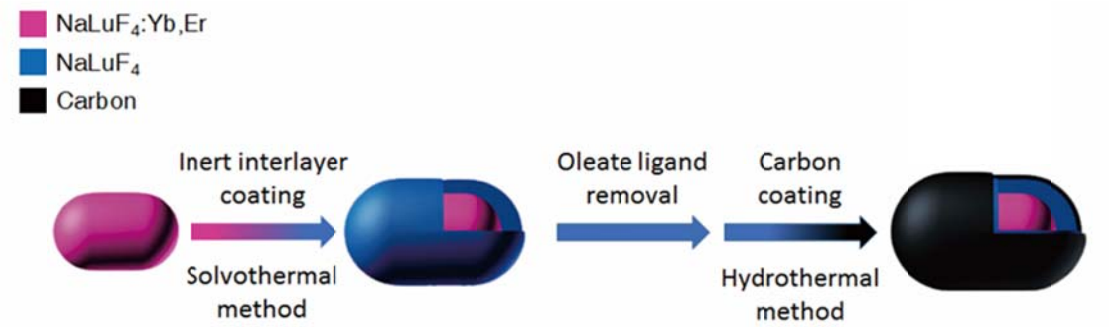

Supplementary Figure 1. Schematic diagram of the synthetic routine of csUCNP@C. Upconversion emissive core of $NaLuF_4$:Yb,Er (UCNPs) were first synthesized by the solvothermal method. Then, an inert interlayer of non-doping $NaLuF_4$ was coated on the upconversion core for the preparation of csUCNPs. After removal of the oleate ligands of csUCNPs, hydrophilic csUCNPs were dispersed into an aqueous solution of glucose for the hydrothermal synthesis of csUCNP@C.

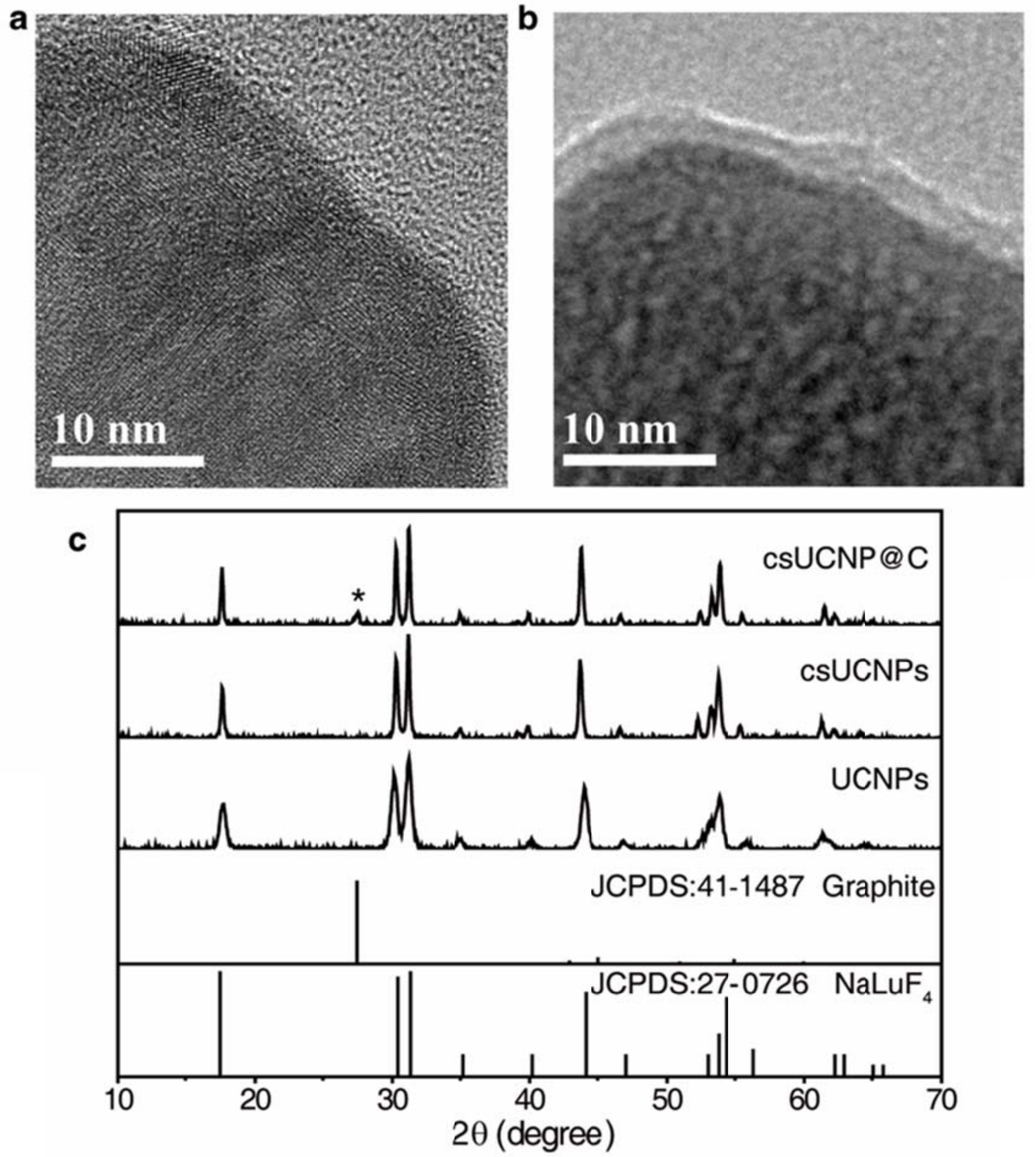

Supplementary Figure 2. Supplementary characterization of morphology and crystalline phase of csUCNP@C. (a) csUCNPs after oleate ligand removal. No obvious residues can be observed on the nanoparticles' surface. **(b)** High resolution TEM images of csUCNP@C from which a thin layer of carbon can be seen on the surface of the nanoparticles. **(c)** XRD patterns of csUCNP@C, csUCNPs and UCNPs. The standard pattern of pure hexagonal $NaLuF_4$ (JPCDs card No. 27-0725) and graphite (JPCDs card No. 41-1487). The graphitic component in csUCNP@C is marked with an asterisk.

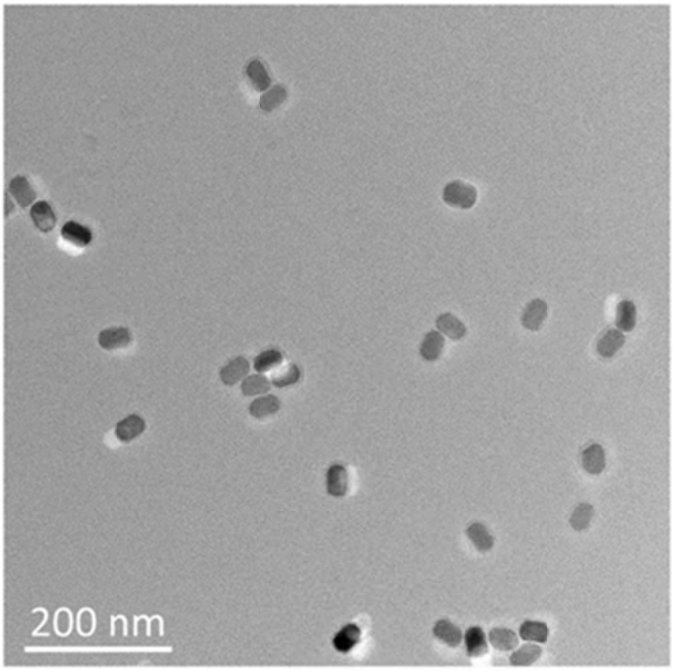

Supplementary Figure 3. Low magnification TEM image of csUCNP@C.

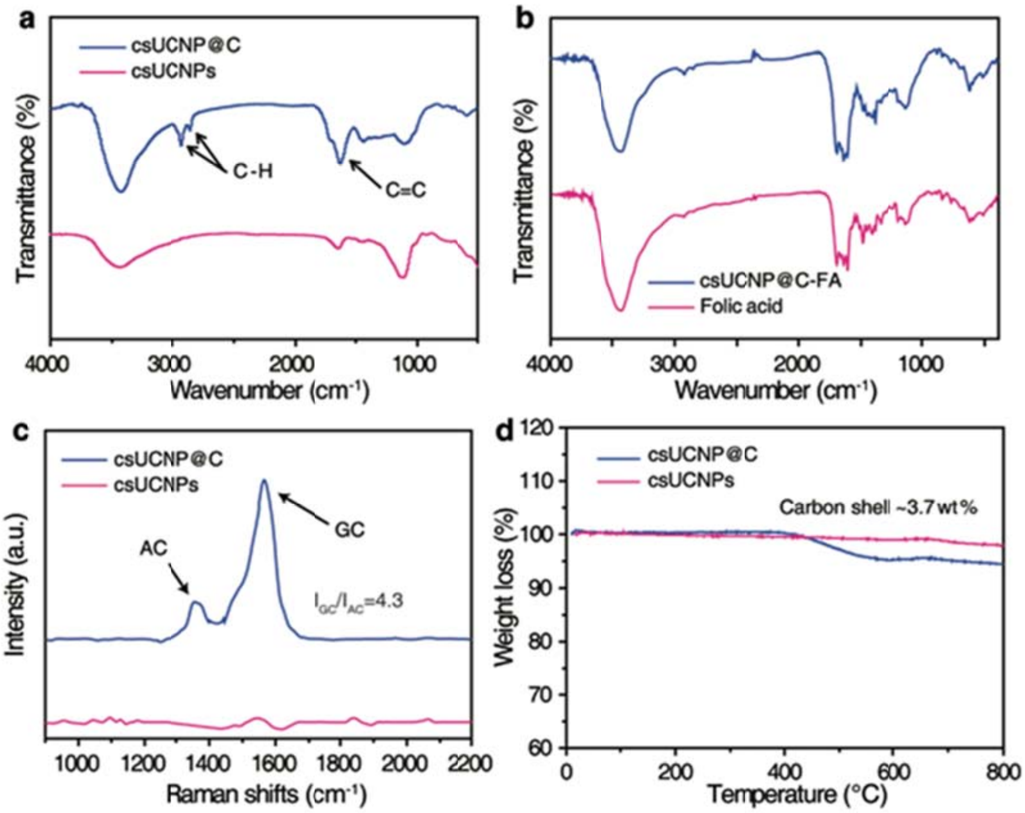

Supplementary Figure 4. Characterization of csUCNP@C. (a) FTIR spectra of csUCNP@C and ligand-free csUCNP after removal of oleic acid. The stretching bands referring to C-H and C=C in csUCNP@C are marked with arrows. **(b)** FTIR spectra of csUCNP@C-FA and folic acid. The characterized peaks of folic acid appeared in the FTIR specturm of csUCNP@C-FA which indicated that the successful conjugation of folic acid on the surface of csUCNP@C. **(c)** Raman spectra of csUCNP@C and csUCNPs. The peaks referring to graphitic carbon (GC) and amorphous carbon (AC) are marked with arrows. The peak intensity ratio of GC and AC (I_{GC}/I_{AC}) in csUCNP@C is 4.3. **(d)** Thermogravimetric analysis (TGA) of csUCNP@C and csUCNP. The weight percentage (wt%) of the carbon shell on csUCNP@C is ~3.7 wt%.

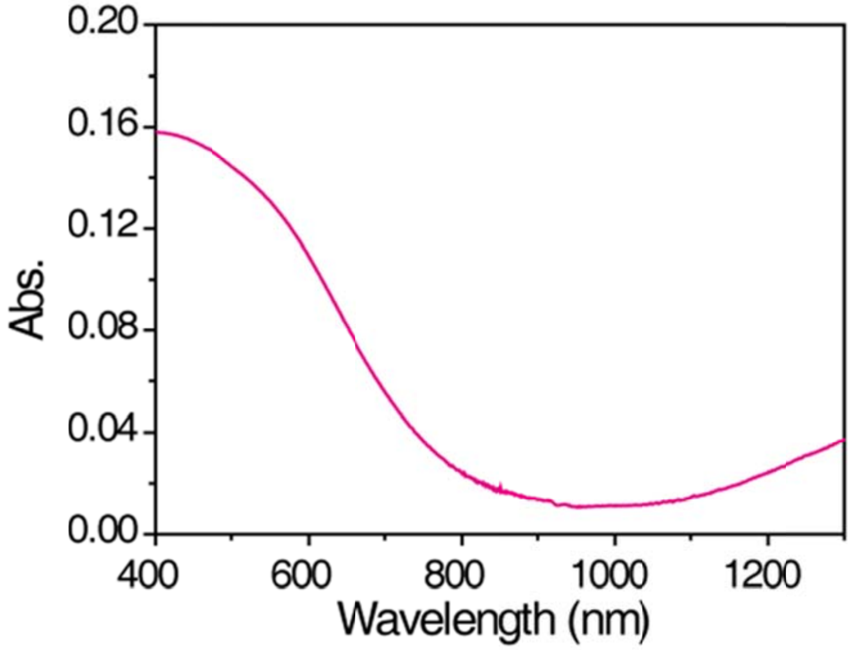

Supplementary Figure 5. UV-NIR absorption spectrum of csUCNP@C aqueous solution.

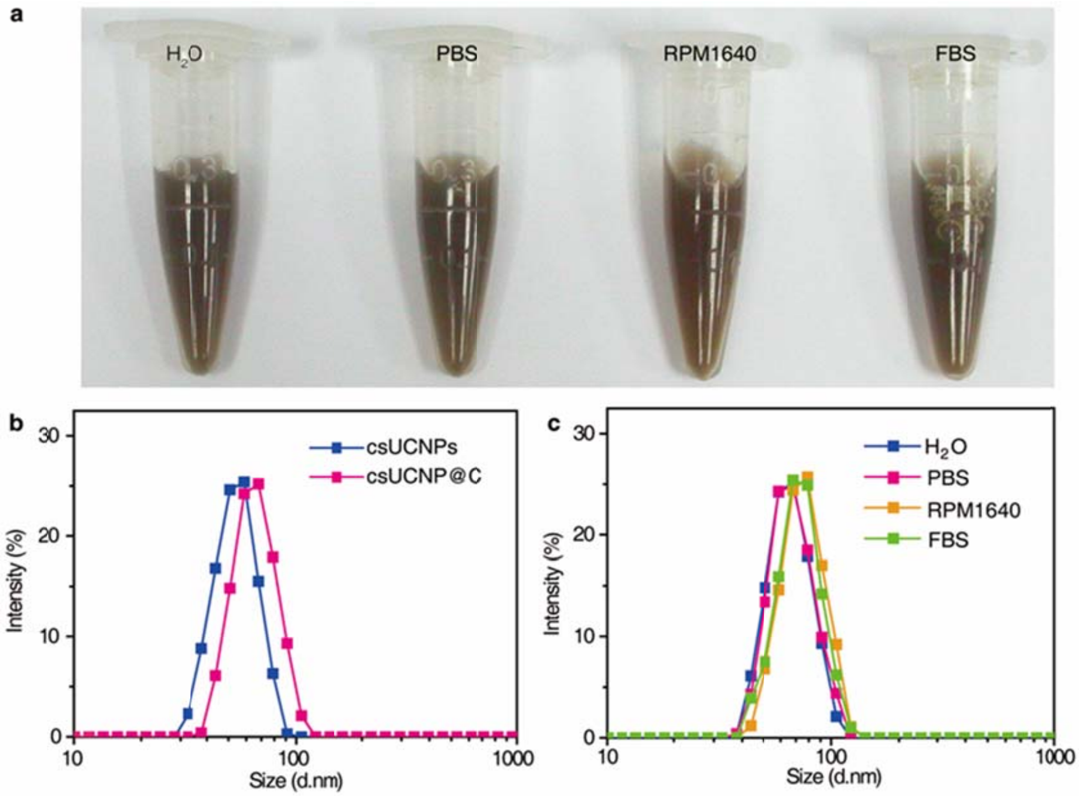

Supplementary Figure 6. Dispersibility of csUCNP@C. (a) In water, phosphate buffered solution (PBS), culture medium and fetal bovine serum (FBS). The as-prepared csUCNP@C can be easily dispersed and were stable in all the above-mentioned aqueous systems. **(b)** Dynamic light scattering of csUCNPs and csUCNP@C. **(c)** The corresponding DLS data in **(a)**.

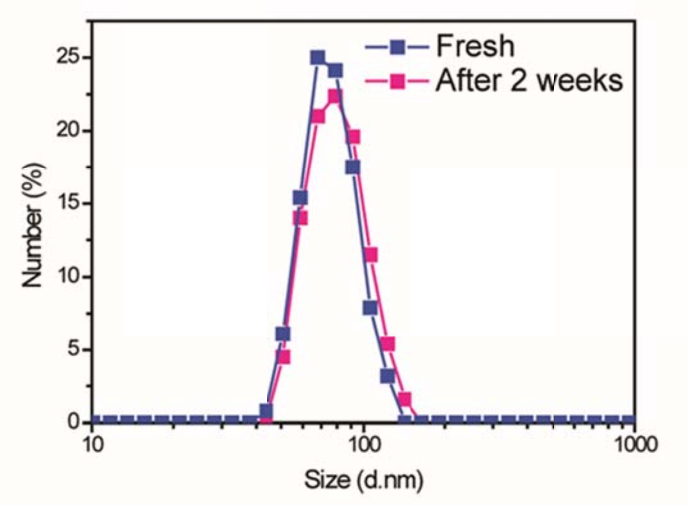

Supplementary Figure 7. Stability of csUCNP@C in aqueous solution. Dynamic light scattering data of csUCNP@C that is freshly prepared and stored for 2 weeks.

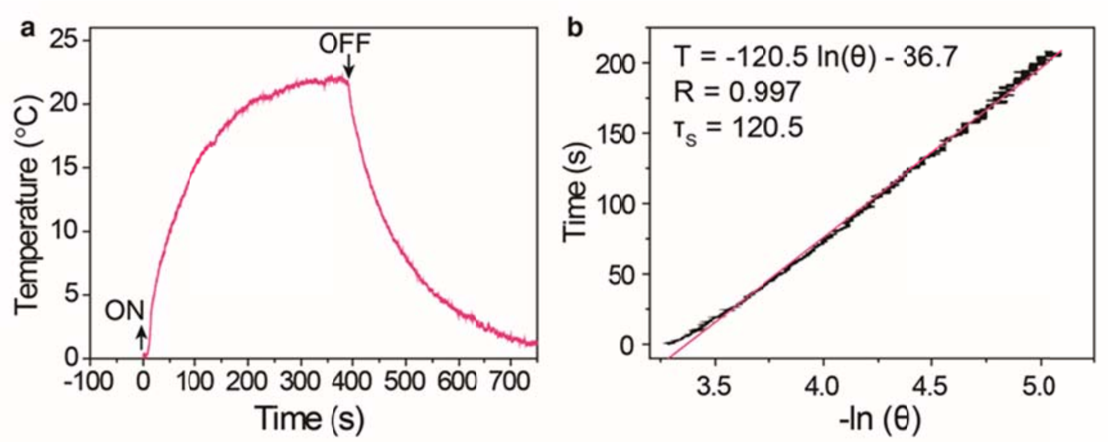

Supplementary Figure 8. (a) Photothermal effect of the irradiation of the aqueous dispersion of csUCNP@C with the 730 nm laser (1 W cm^{-2}), in which the irradiation lasted for 400 s, and then the laser is shut off. **(b)** Time constant for heat transfer from the system is determined to be τ_s = 120.5 s by applying the linear time data from the cooling period (after 400 s) versus negative natural logarithm of driving force temperature, which is obtained from the cooling stage in **(a)**.

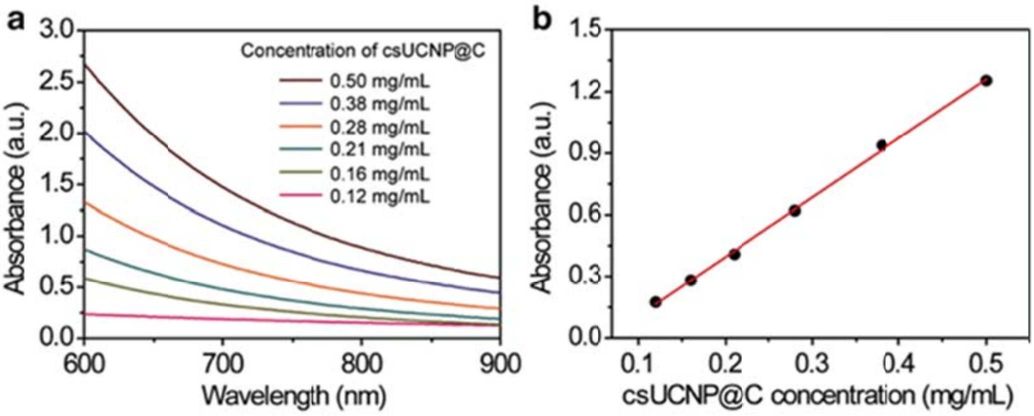

Supplementary Figure 9. (a) Room temperature UV-vis absorbance spectra of csUCNP@C aqueous solutions with different concentrations. **(b)** A linear relation for the optical absorbance at 730 nm as a function of carbon shell concentration on csUCNP@C.

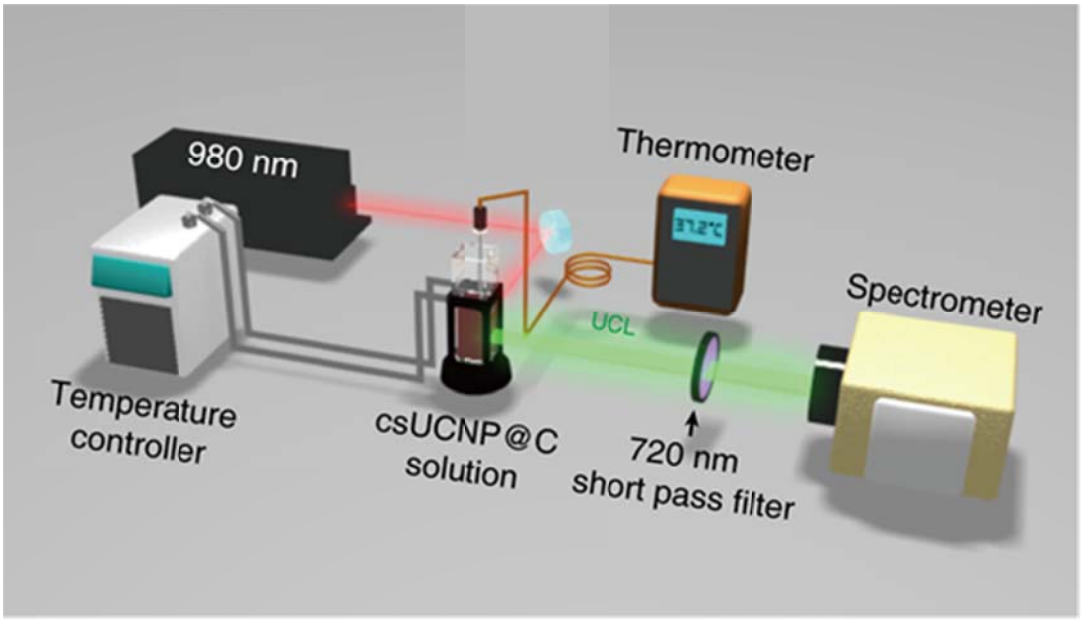

Supplementary Figure 10. Schematic diagram of the set-up for temperature calibration. By heating the solution of csUCNP@C, the UCL peak intensity at 525 nm was enhanced correspondingly with rising temperature (Fig. 2e). The dependence of $ln(I_{525}/I_{545})$ on the inverse temperature, $(1/T)$, showing a linear behavior (Fig. 2f), is well fitted as $ln(I_{525}/I_{545}) = 1.083 - 0.844 \times (1/T)$ (T given in K). The fitted curve was then utilized as calibration to determine the real-time eigen temperature of csUCNP@C under photothermal irradiation at 730 nm.

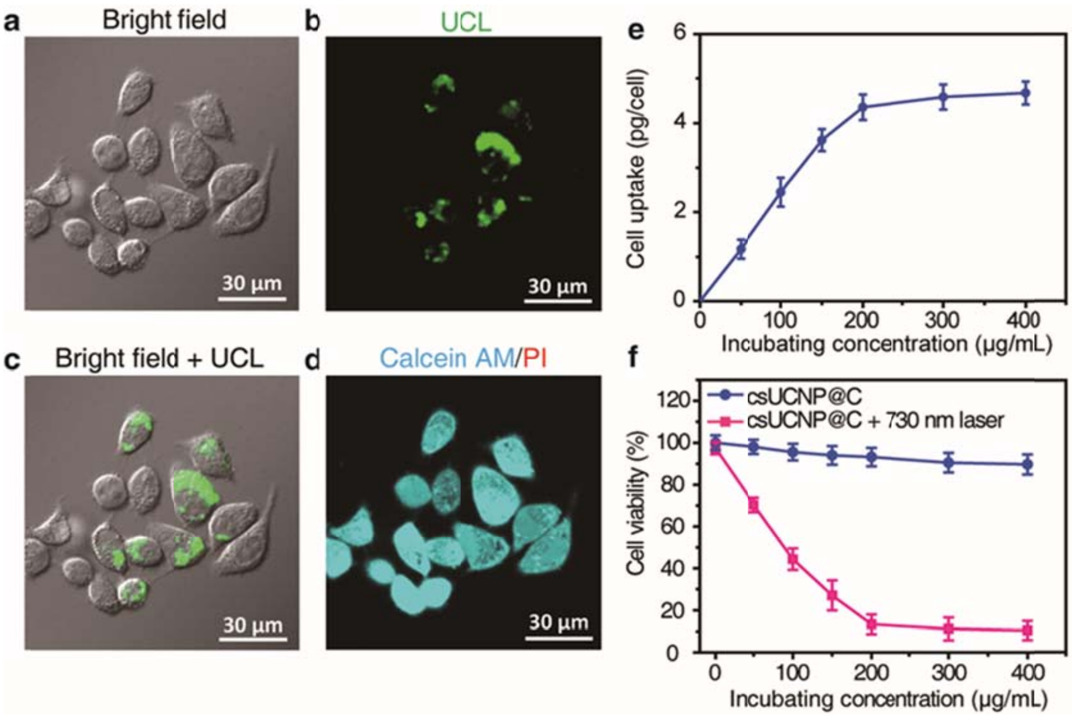

Supplementary Figure 11. Cell viability of mixed cells before and after photothermal therapy. (a) Bright-field image of csUCNP@C-labeled cells and non-treated HeLa cells. **(b)** UCL image of the mixed cells. UCL signals were collected from 500 to 580 nm under 980 nm excitation. **(c)** Overlay image of panels **(a)** and **(b)**. **(d)** Calcein AM and PI double staining of the mixed cells before photothermal therapy. The mixed cells are all stained with calcein AM, indicating that the cells are all alive before photothermal therapy. Thus csUCNP@C has good cell compatibility. Calcein AM signals were collected from 500-560 nm under 488 nm laser excitation. PI signals were collected from 600 to 680 nm under 633 nm laser excitation. **(e)** Cell uptake of csUCNP@C with different incubation concentrations of csUCNP@C. The amount of csUCNP@C is represented as the average uptake of each cell. Average values of cell uptake under different incubating concentration were given based on 3 sets of parallel test. Error bars were defined as standard deviation. **(f)** Methyl thiazolyl tetrazolium (MTT) assays of HeLa cells incubated with csUCNP@C at different concentrations (0, 50, 100, 150, 200, 300, or 400 μg ml^{-1}. The corresponding uptake amounts are 1.1, 2.5, 3.6, 4.4, 4.6 and 4.7 pg per cell) for 24 h and the counterpart was treated with 730 nm laser irradiation for 5 min at 0.3 W cm^{-2}. Cell viability of 90% even at the high dosage of csUCNP@C (400 μg ml^{-1}) was observed after 24 h, indicating that csUCNP@C possesses good cell viability. Under 730 nm laser irradiation, cell viability decreased substantially and began to reach a minimum level at 200 μg ml^{-1} demonstrating the excellent photothermal effect of csUCNP@C. Average values of cell viability under different incubating

concentration were given based on 3 sets of parallel test. Error bars were defined as standard deviation.

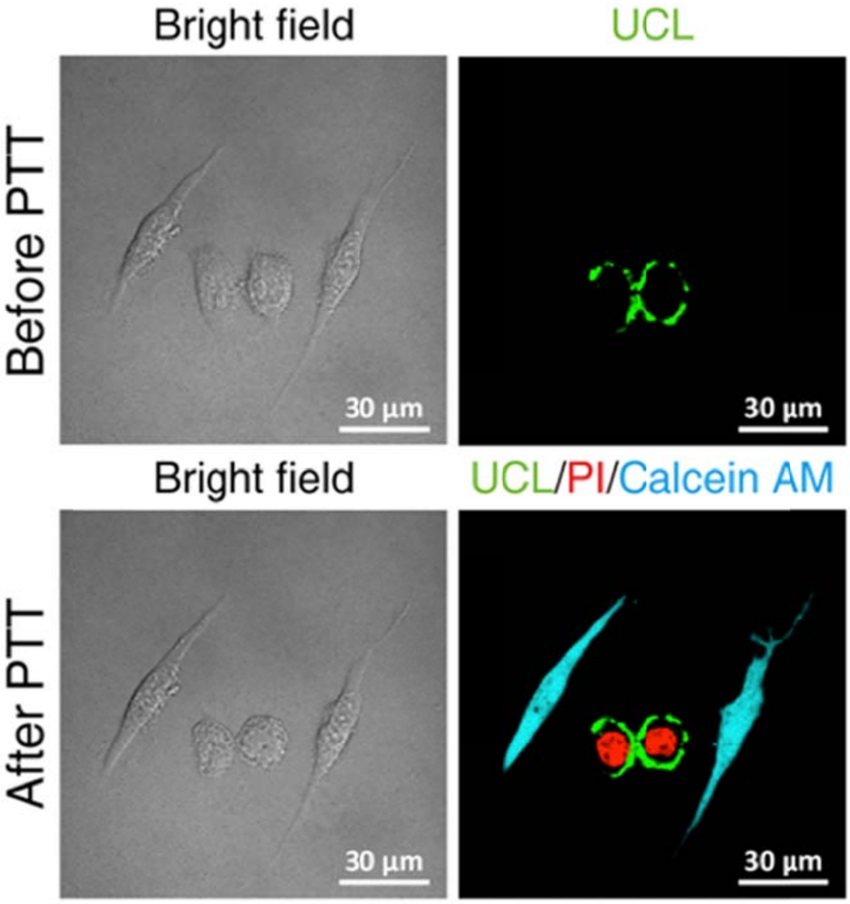

Supplementary Figure 12. Photothermal therapy of csUCNP@C labeled HeLa cells and non-labeled C2C12 cells under 730 nm laser irradiation at 0.3 W cm^{-2} for 5 min. HeLa cells labeled with csUCNP@C showed a strong UCL signal in the cytoplasm (green). The signal is collected in the wavelength region of 500-580 nm. Calcein AM (cyan) & PI (red) double staining showed that only the HeLa cells labeled with csUCNP@C were dead.

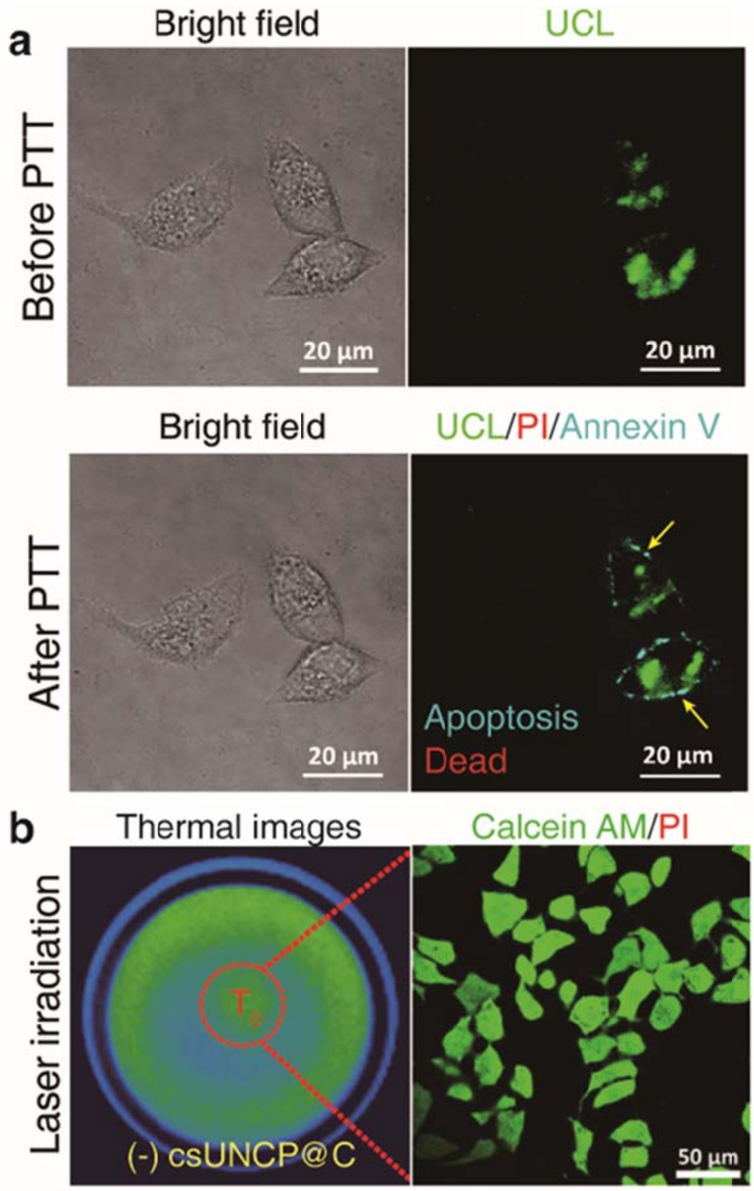

Supplementary Figure 13. High-accuracy photothermal treatment at a low apparent temperature. **(a)** Mixed cells were treated with 730 nm laser irradiation at 0.2 W cm^{-2} for 5 min. Annexin V-FITC and PI were applied to the cells at 30 min after PTT to determine apoptosis (Annexin V-FITC, cyan) and necrosis (PI, red). There was no obvious change in cell morphology after photothermal treatment, but the membrane of csUCNP@C-labeled HeLa cells (UCL, green) was stained by Annexin V-FITC (cyan), marked by yellow arrows, indicating an early stage of apoptosis. **(b)** Non-labeled cells under 730 nm laser irradiation (0.3 W cm^{-2}). Cells exposed to laser spot were marked with red circles. The maximum temperature in the laser spot was set as T_0. Cells in the laser spot were all alive (Calcein AM, green), indicating that the weak heating effect of the laser did not have any damaging effects.

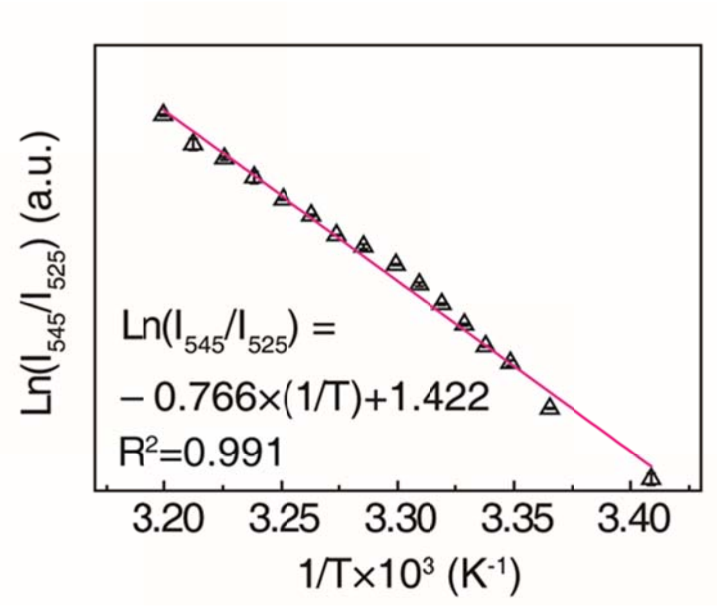

Supplementary Figure 14. Temperature calibration in cells. A plot of $\ln(I_{525}/I_{545})$ *vs* 1/T to calibrate the thermometric scale for csUCNP@C in HeLa cells. Average values of I_{525}/I_{545} under different temperature were given to fit the calibration curve based on 3 times measurements of UCL spectrum. Error bars were defined as standard deviation.

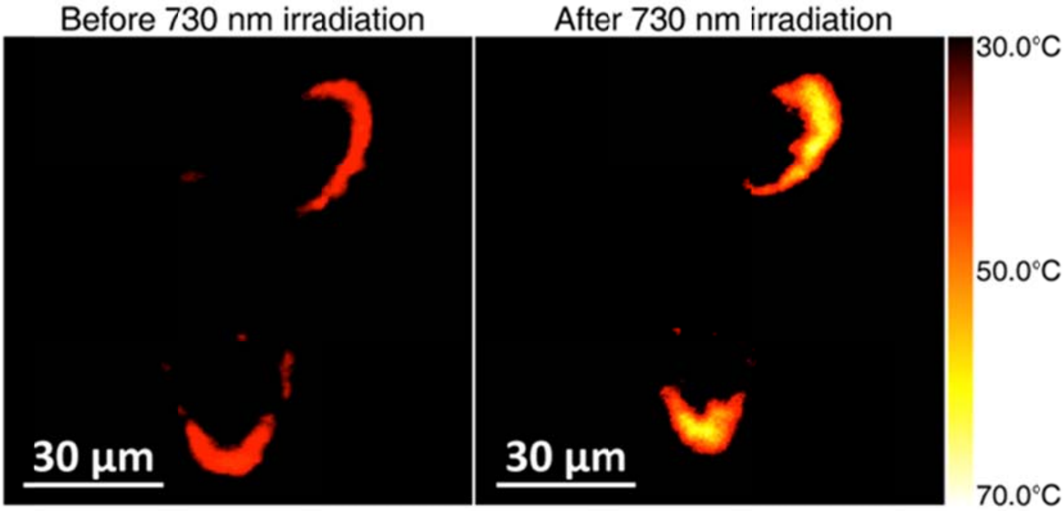

Supplementary Figure 15. Eigen temperature in cells. Eigen temperature mapping of csUCNP@C labeled HeLa cells before and after 730 nm laser irradiation (5 min, 0.3 W cm^{-2}). Temperature mapping of HeLa cells were acquired according to the thermal equilibrium : $(I_{545}) / (I_{525}) = C \exp(- \Delta E / kT)$ where I_{545} and I_{525} are the UCL emission in the wavelength region of 535-580 nm and 515-535 nm, respectively. After 730 nm irradiation, $(I_{545}) / (I_{525})$ decreased and correspondingly eigen temperature of the cells increased.

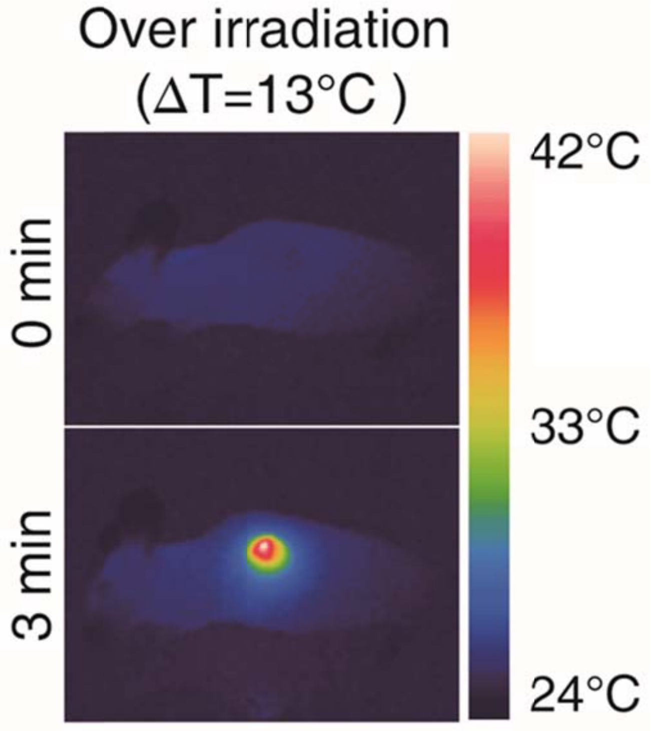

Supplementary Figure 16. Photothermal therapy at high apparent temperature. Thermal images of nude mice with csUCNP@C-labeled HeLa cell tumors under 730 nm irradiation (0.8 W cm^{-2}). ΔT = (maximum of the apparent temperature at tumor site under laser irradiation) – (maximum of the apparent temperature at tumor site before laser irradiation). The apparent temperature is controlled at 42°C (ΔT = 13°C) which is a critical temperature for current PTT. The corresponding H&E histologic section of the border of tumor and normal fat tissue indicate that the adipocytes (Ad) in normal fat tissue suffered extreme damage.

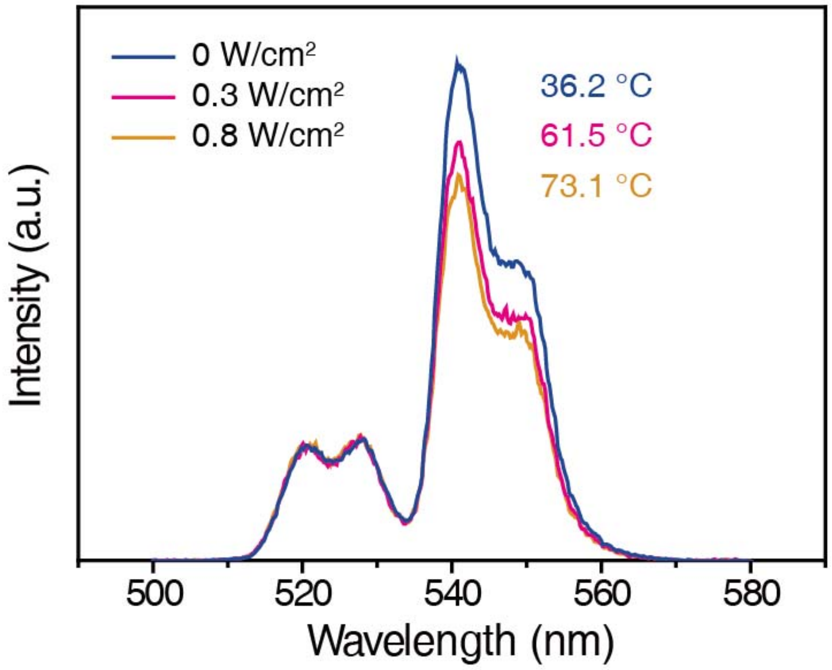

Supplementary Figure 17. Eigen temperature monitoring in tumor-bearing mice. Upconversion luminescence spectra of csUCNP@C (500-580 nm) in tumor-bearing mice detected by fiber-optic spectrometer under different 730 nm laser power density (0, 0.3 and 0.8 W cm^{-2}) for 3 min. Eigen temperature of csUCNP@C under different 730 nm laser power density irradiation were calculated by the upconversion luminescence spectra according to the calibration curve in tissue phantom. Under 730 nm laser irradiation at 0.3 W cm^{-2}, the eigen temperature was sufficient to kill cancer cells, which was higher than the eigen temperature in cell experiment (Fig. 3 and Supplementary Fig. 15). Under 730 nm laser irradiation at 0.8 W cm^{-2}, the apparent temperature reached to a high level and caused a massive heat conduction (Supplementary Fig. 16).

a

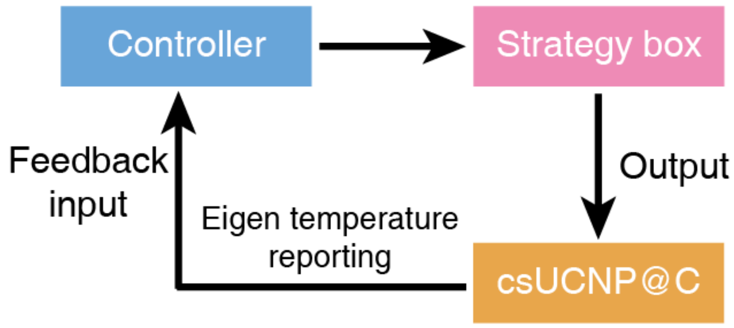

b

Treatment strategy	1#	2#
Laser power density (W/cm^2)	0.3	0.8
Eigen temperature (°C)	61.5	73.1
Apparent temperature elevation (°C)	1.5	13
Cell ablation	+	+
Massive heat conduction	–	+
Facile therapy judgment	+	–

Supplementary Figure 18. Demonstration of temperature feedback photothermal treatment. (a) Model of temperature feedback photothermal treatment system. In this model, controller will make decision on the treatment strategy. Strategy box stores a series of photothermal therapy strategies for selection. csUCNP@C receives the strategy output and is served as both photothermal agent and eigen temperature reporter for feedback signal input. **(b)** Evaluation of the two treatment strategies (1# and 2#) in this work according to the principle model in **(a)**. These two treatment strategies (1# and 2#) constitute a strategy box, upconversion luminescence signal of csUCNP@C reports the eigen temperature to help the controller (In this work, controller is the experimenter) to adopt what kind of treatment strategy. The treatment strategy 1# with 730 nm laser irradiation at 0.3 W cm^{-2} has a high eigen temperature and a limited heat conduction, so that it can be determined as facile therapy strategy.

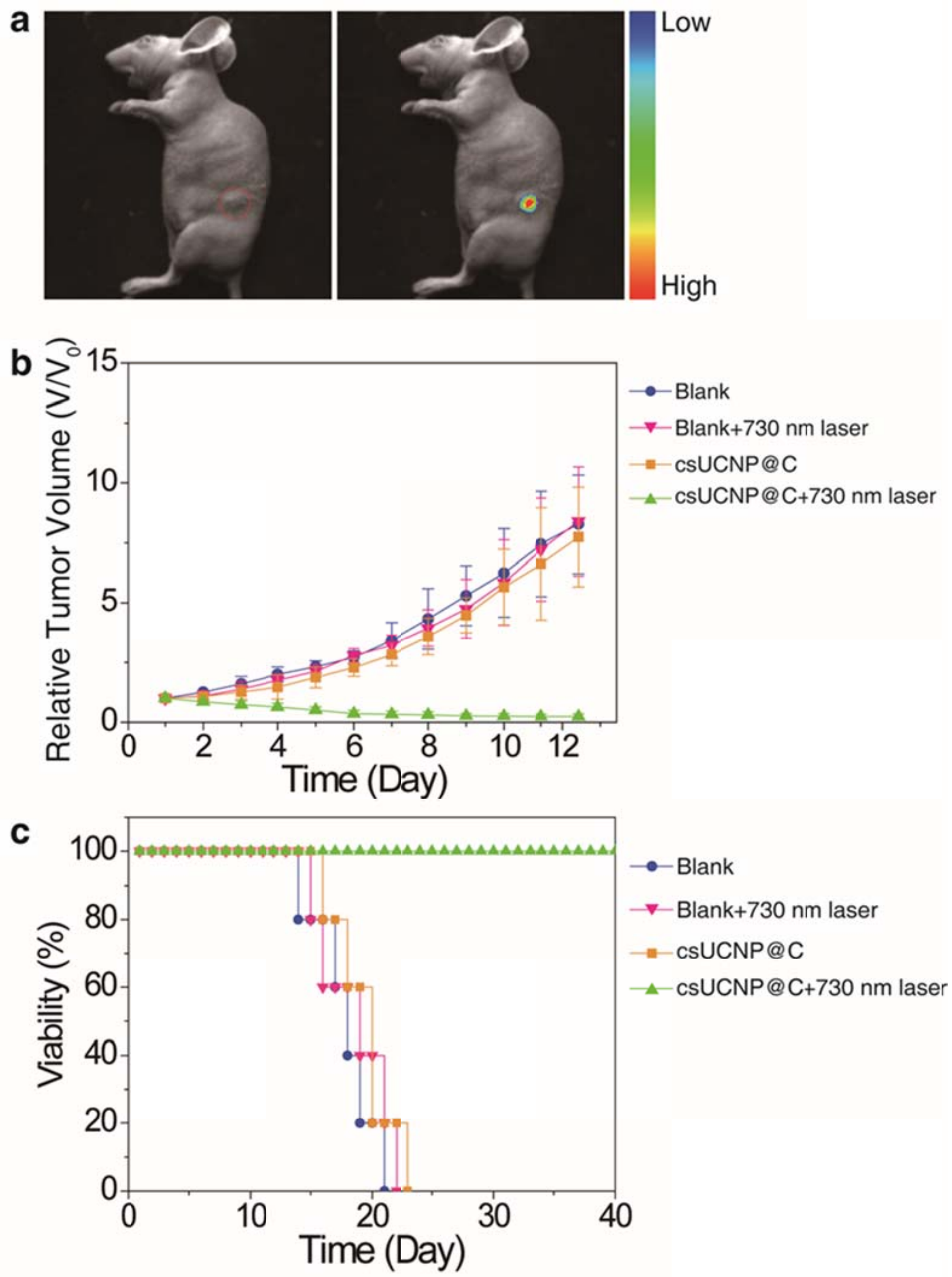

Supplementary Figure 19. Photothermal therapy of tumor-bearing mice. (a) *In vivo* UCL imaging of a nude mouse bearing a csUCNP@C-labeled HeLa cell tumor. UCL signals were collected by using a 720 nm short pass filter. **(b)** Growth of tumors in different groups of mice (five mice in each group) after photothermal treatment. The relative tumor volumes were normalized to their initial sizes. Average values of tumour volume of each group were based on the measurement of the tumors in five mice. Error bars were defined as standard deviation. **(c)** Survival curves of mice after various treatments as indicated in **(b)**.

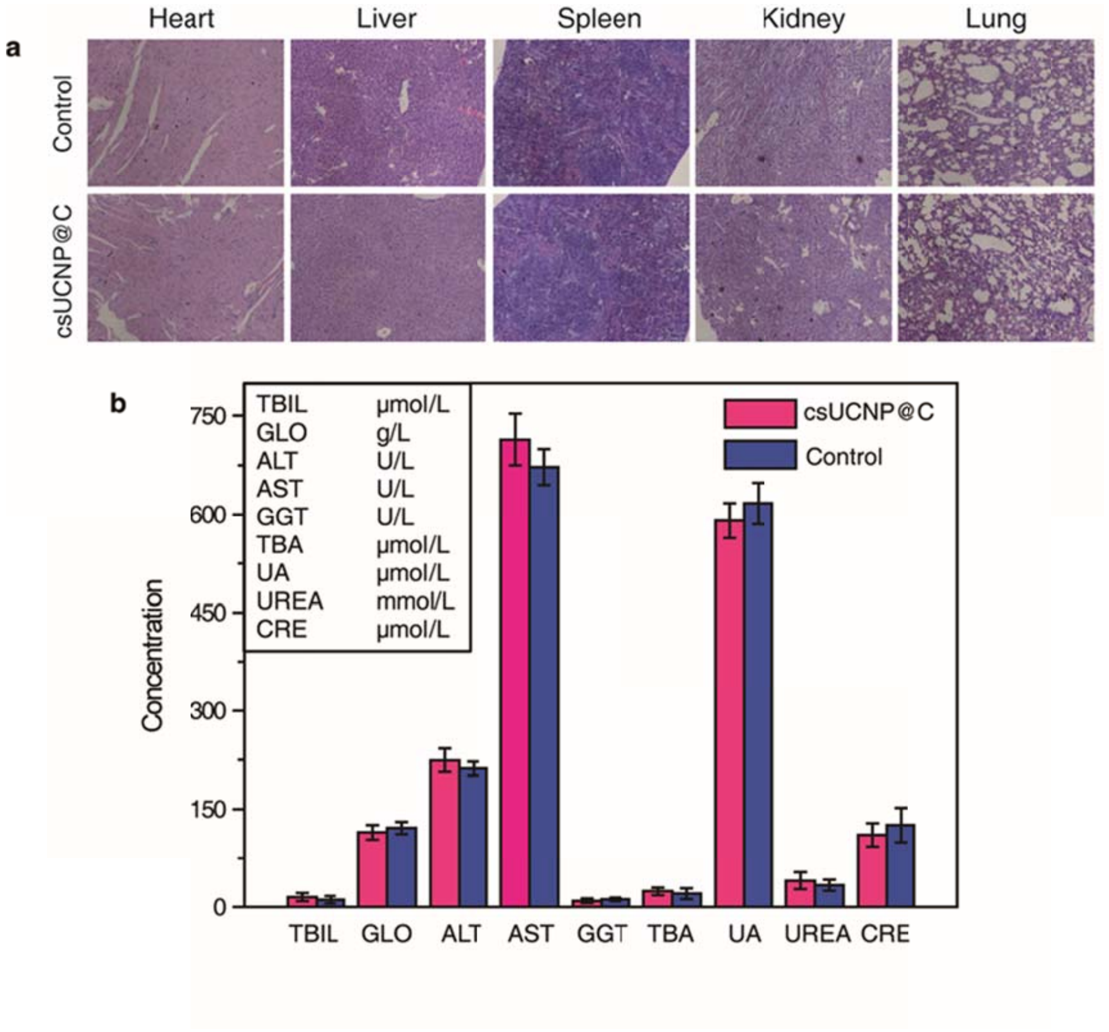

Supplementary Figure 20. Biotoxicity of folic acid modified csUCNP@C (FA-csUCNP@C). (a) Histological changes in the heart, liver, spleen, kidney and lung of nude mice one week after intravenous injection of csUCNP@C in PBS solution. The organs are stained with hematoxylin and eosin (H&E) and are observed under an optical microscope at 100 × magnification. **(b)** Serum biochemistry assays of the mice injected with FA-csUCNP@C. Untreated mice acted as the control group. Serum biochemistry and histological section assays of mice at 7 days post intravenous injection of FA-csUCNP@C showed no appreciable abnormalities when compared with the untreated group, suggesting no evident toxic effects *in vivo* within one week of FA-csUCNP@C. Each assay index was the average value based on the blood samples of three mice. Error bars were defined as standard deviation.

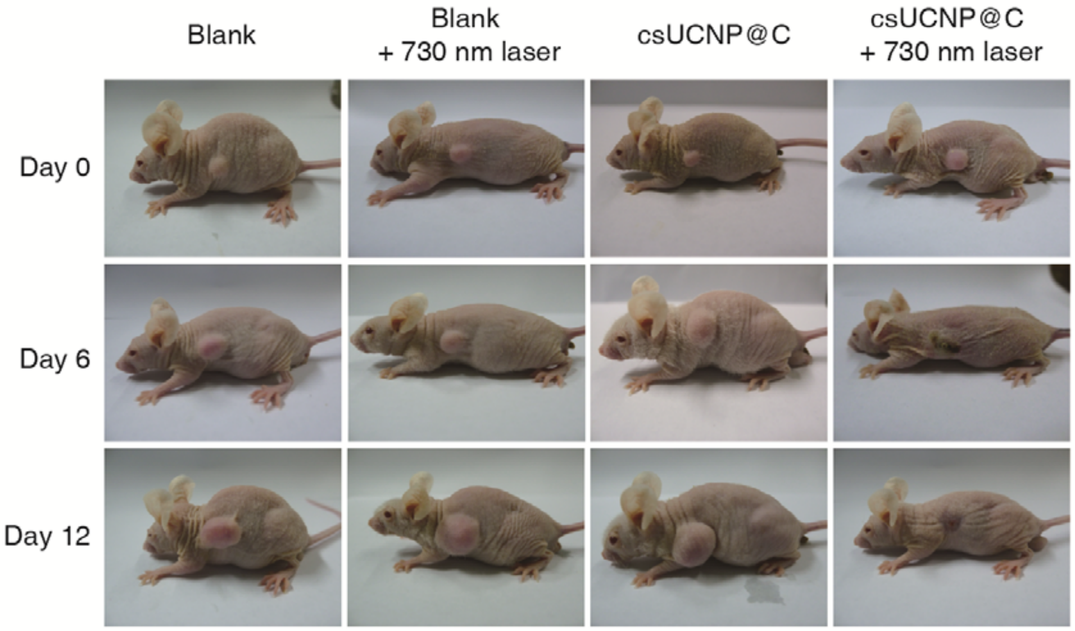

Supplementary Figure 21. Photothermal therapy *in vivo*. Representative photos of tumor-bearing nude mice before and after treatment. The tumors in the mice injected with csUCNP@C with 730 nm irradiation (0.3 W cm^{-2}) shrank, while the other groups (blank, 730 nm laser alone, and csUCNP@C alone) showed a weak effect on tumor growth.

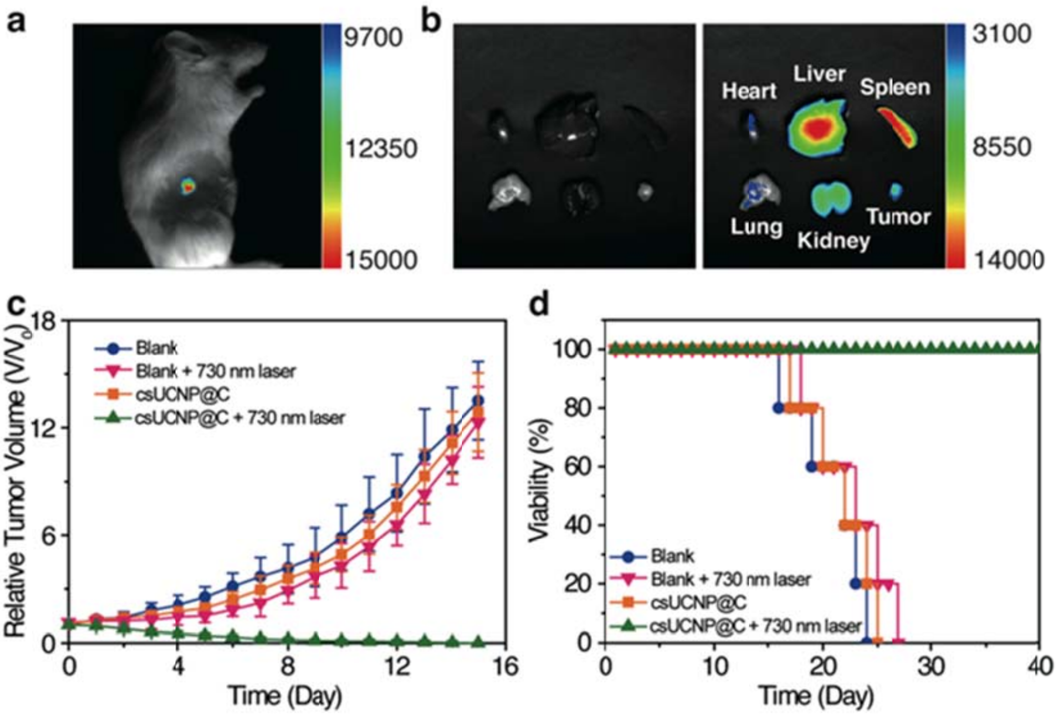

Supplementary Figure 22. Photothermal therapy of tumor-bearing Balb/c mice. (a) *In vivo* UCL imaging of HeLa tumor-bearing Balb/c mice 2 h after intravenous injection of FA-csUCNP@C (2 mg ml^{-1}, 200 μL). **(b)** *Ex vivo* UCL image of the major organs of the injected mice (heart, liver, spleen, lung, kidney and tumor). UCL signals were collected by using a 720 nm short pass filter. **(c)** Growth of tumors in different groups of Balb/c mice (five mice in each group) after photothermal treatment. The relative tumor volumes were normalized to their initial sizes. Average values of tumour volume of each group were based on the measurement of the tumors in five mice. Error bars were defined as standard deviation. **(d)** Survival curves of mice after various treatments as indicated in **(c)**.

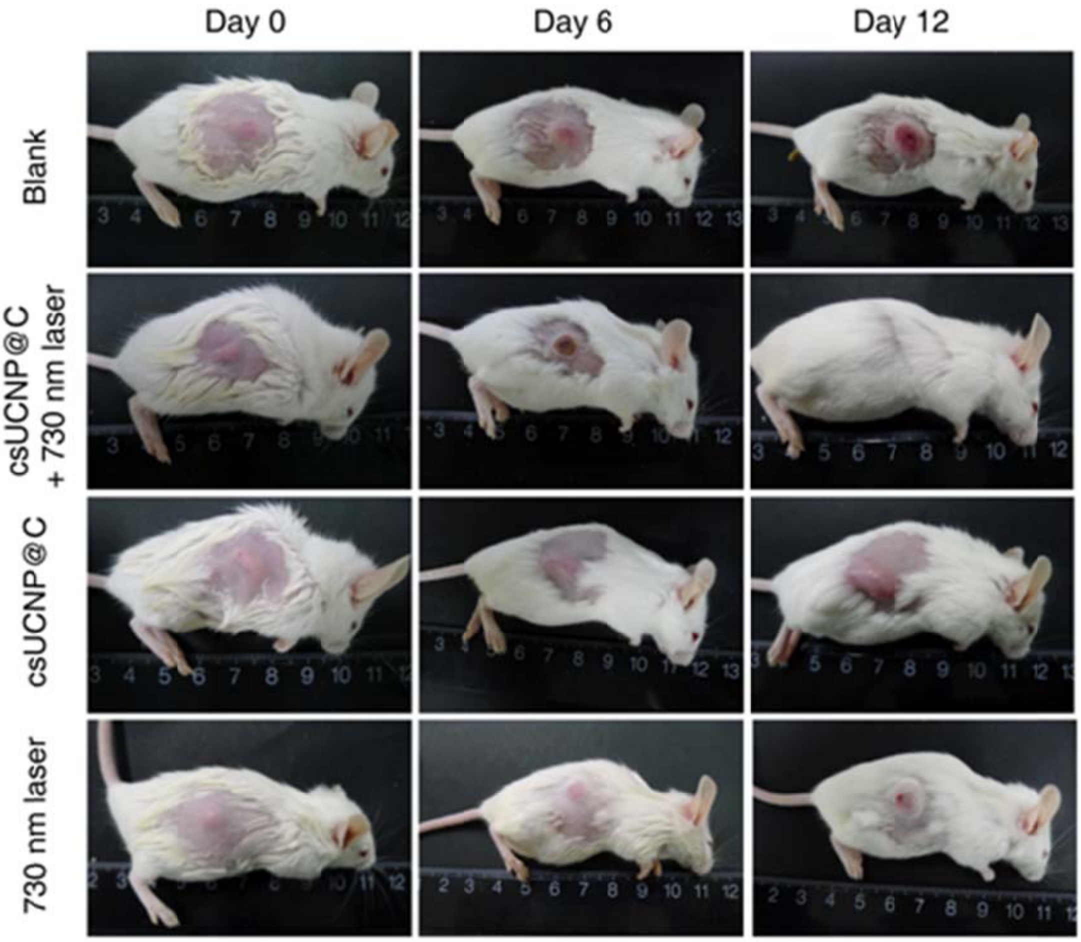

Supplementary Figure 23. Photothermal therapy *in vivo*. Representative photos of tumor-bearing Balb/c mice before and after treatment. The tumors in the mice injected with csUCNP@C with 730 nm irradiation (0.3 W cm^{-2}) shrank, while the other groups (blank, 730 nm laser alone, and csUCNP@C alone) showed a weak effect on tumor growth.

Supplementary Table 1. Tumor size changes in nude mice with photothermal treatment.

	Blank		Blank+730 nm laser		csUCNP@C		csUCNP@C +730 nm laser	
Day	Tumour length (mm)	Tumour width (mm)	Tumour length (mm)	Tumour width (mm)	Tumour length (mm)	Tumour width (mm)	Tumour length (mm)	Tumour width (mm)
1	6.12	6.06	6.12	6.12	6.12	6.06	6.12	6.06
	6.06	6.06	6.12	6.06	6.06	6.06	5.94	5.88
	6.18	6.12	6.12	6.06	6.18	6	6.06	6.06
	6.18	6.12	6.06	5.94	6.06	5.94	6	5.94
	6.06	5.94	6	6.06	6.06	5.88	6.12	6.06
2	6.78	6.72	6.3	6.18	6.24	6.18	5.82	5.76
	6.72	6.66	6.24	6.18	6.18	6.18	5.64	5.58
	6.66	6.66	6.24	6.18	6.18	6.12	5.58	5.52
	6.36	6.3	6.18	6.06	6.12	6.06	5.64	5.64
	6.3	6.24	6.12	6.12	6.12	6.06	5.58	5.45
3	7.44	7.32	7.14	7.08	6.54	6.48	5.58	5.4
	7.38	7.32	7.02	6.96	6.42	6.36	5.52	5.22
	7.14	7.02	6.66	6.48	6.36	6.36	5.04	4.92
	6.66	6.54	6.3	6.18	6.36	6.3	5.22	5.28
	6.48	6.36	6.24	6.24	6.3	6.3	5.1	5.04
4	8.16	8.04	7.68	7.68	7.26	7.08	5.28	5.12
	7.74	7.68	7.56	7.5	7.08	7.02	5.16	4.92
	7.62	7.62	6.96	6.84	6.66	6.6	4.8	4.68
	7.26	7.08	6.9	6.84	6.54	6.54	5.04	4.98
	6.84	6.78	6.9	6.78	6.48	6.42	4.8	4.86
5	8.52	8.4	8.22	8.1	8.46	8.34	5.1	5.04
	8.16	8.04	8.04	7.92	7.56	7.62	4.92	4.92
	7.98	7.92	7.74	7.62	6.96	6.96	4.68	4.38
	7.98	7.86	7.68	7.62	6.78	6.72	4.92	4.8
	7.62	7.5	7.38	7.26	6.72	6.54	4.68	4.68
6	8.76	8.7	8.82	8.64	8.76	8.7	3.52	3.31
	8.52	8.46	8.7	8.58	8.04	8.1	3.84	3.6
	8.22	8.16	8.52	8.34	7.56	7.5	3.84	3.66
	8.1	8.1	8.16	8.04	7.44	7.38	1.78	1.48
	8.04	7.98	7.86	7.74	7.38	7.32	3.36	3.18

7	9.9	9.84	9.36	9.3	9.42	9.36	1.64	1.48
	9.72	9.6	9.06	8.88	8.52	8.34	1.52	1.06
	8.58	8.58	8.64	8.52	8.1	7.98	2.16	2.04
	8.46	8.4	8.82	8.7	8.04	7.92	0.42	0.37
	8.22	8.16	8.22	8.04	7.92	7.86	1.27	1.18
8	10.92	10.8	10.38	10.32	10.44	10.26	1.36	1.14
	10.8	10.8	9.9	9.54	9.36	9.18	0.52	0.35
	9.18	9.18	9.24	9.18	8.7	8.58	1.44	1.26
	8.64	8.64	9	8.94	8.46	8.34	0.24	0.13
	8.46	8.28	8.46	8.28	8.34	8.28	0.43	0.32
9	11.46	11.28	11.52	11.22	10.98	10.8	0.52	0.46
	11.52	11.4	10.44	10.32	10.38	10.26	0.19	0.14
	10.26	10.08	9.78	9.54	9.06	9	0.48	0.36
	9.66	9.54	9.42	9.36	9.3	9.12	0.07	0.05
	9.24	9.06	8.94	8.76	9.24	8.94	0.18	0.11
10	12.12	11.88	12.6	12.24	11.94	11.82	0.24	0.16
	12.3	12.21	11.4	11.34	11.22	11.16	0.06	0.04
	10.74	10.68	10.2	9.84	9.96	9.84	0.24	0.13
	9.9	9.84	10.02	9.84	9.84	9.78	0	0
	9.54	9.54	9.36	9.3	9.66	9.48	0.08	0.06
11	13.2	13.14	13.26	13.08	12.9	12.54	0.12	0.07
	12.72	12.66	12.42	12.3	12.54	12.42	0	0
	10.86	10.86	10.86	10.8	9.96	9.84	0.11	0.05
	10.68	10.56	10.92	10.74	10.2	10.14	0	0
	10.56	10.5	10.14	10.08	10.14	9.84	0	0
12	13.56	13.32	13.8	13.62	13.32	12.9	0	0
	13.02	12.96	13.02	12.96	12.9	12.72	0	0
	11.4	11.4	11.58	11.28	11.34	11.22	0	0
	11.22	11.1	11.28	11.34	10.53	10.38	0	0
	11.04	10.98	10.8	10.68	10.5	10.68	0	0

Supplementary Table 2. Tumor size changes in Balb/c mice with photothermal treatment.

	Blank		Blank+730 nm laser		csUCNP@C		csUCNP@C +730 nm laser	
Day	Tumour length (mm)	Tumour width (mm)	Tumour length (mm)	Tumour width (mm)	Tumour length (mm)	Tumour width (mm)	Tumour length (mm)	Tumour width (mm)
0	6.18	6.12	6.12	6.06	6.12	6.12	6.18	6.12
	6.12	6.06	6.18	6.12	6	6.06	6.12	6.12
	6.12	6.06	6.06	6.06	6.12	6.06	6.06	5.94
	6.12	6.12	6.18	6.12	6.12	6.06	6.06	6.06
	6.12	6.06	6.12	6.06	6.06	5.94	6.18	6.12
1	6.36	6.36	6.42	6.36	6.48	6.42	5.88	5.82
	6.18	6.18	6.3	6.18	6.24	6.12	5.76	5.7
	6.3	6.24	6.42	6.36	6.3	6.12	5.76	5.58
	6.3	6.18	6.36	6.18	6.3	6.18	5.88	5.88
	6.42	6.3	6.42	6.3	6.3	6.24	5.82	5.82
2	6.84	6.78	6.48	6.42	6.72	6.6	5.64	5.58
	6.84	6.72	6.42	6.24	6.54	6.48	5.52	5.46
	6.72	6.6	6.48	6.36	6.78	6.54	5.52	5.34
	6.66	6.54	6.48	6.42	6.72	6.6	5.64	5.58
	6.6	6.54	6.36	6.24	6.54	6.54	5.34	5.46
3	7.74	7.68	6.84	6.72	7.38	7.26	5.34	5.4
	7.68	7.56	6.72	6.54	7.2	7.14	5.16	5.04
	7.56	7.5	6.72	6.66	7.14	7.02	5.1	4.98
	7.02	6.96	6.78	6.66	7.02	6.96	5.1	5.04
	6.96	6.72	6.48	6.42	6.72	6.72	5.04	4.92
4	8.64	8.58	6.96	6.9	7.62	7.68	4.74	5.1
	8.28	8.22	7.02	6.78	7.56	7.44	4.62	4.56
	7.86	7.74	6.72	6.66	7.68	7.5	4.44	4.68
	7.5	7.26	6.84	6.78	7.44	7.26	4.5	4.38
	7.32	7.2	6.6	6.48	6.96	6.96	4.56	4.38
5	9.36	8.94	7.44	7.44	8.1	7.98	4.5	4.62
	8.82	8.7	7.44	7.2	7.74	7.68	4.38	4.44
	8.22	8.1	6.9	6.84	7.98	7.68	4.2	4.32
	7.86	7.74	6.96	6.9	7.68	7.56	4.2	4.14
	7.56	7.56	6.84	6.72	7.44	7.44	4.02	4.08

6	10.08	9.84	8.04	7.98	8.94	8.82	4.26	4.2
	9.42	9.3	7.86	7.74	8.22	8.04	3.9	3.84
	8.76	8.64	7.08	7.02	8.7	8.52	3.72	3.66
	8.28	8.16	7.14	7.02	8.04	7.86	3.84	3.48
	8.1	7.98	7.02	6.9	7.62	7.56	3.12	3.36
7	10.56	10.5	8.88	8.82	9.48	9.36	3.66	3.6
	9.96	9.9	8.82	8.7	8.58	8.88	3.12	2.88
	9.06	9	7.62	7.56	9.18	8.88	3	2.82
	8.46	8.34	7.44	7.26	8.34	8.4	3.42	2.7
	8.4	8.34	7.38	7.14	7.92	7.68	2.46	2.4
8	10.98	10.92	9.78	9.66	10.14	9.96	3.6	3.48
	10.38	10.32	9.42	9.36	9.3	9.18	2.58	2.7
	9.12	9.06	8.46	8.16	9.42	9.3	2.64	2.58
	8.76	8.64	8.04	7.92	8.7	8.76	3.06	2.46
	8.7	8.58	8.22	8.1	8.46	8.34	2.28	2.22
9	11.7	11.4	10.44	10.38	10.62	10.5	3.42	3.18
	11.04	10.92	10.38	10.32	10.08	9.96	2.52	2.46
	9.72	9.6	8.88	8.7	9.96	9.78	2.1	2.4
	9.24	8.94	8.34	8.28	8.94	8.88	2.58	2.16
	9.42	9.18	8.46	8.28	8.58	8.52	2.04	1.86
10	12.42	12.36	10.98	10.8	11.28	11.16	2.4	2.28
	11.76	11.64	10.86	10.8	10.74	10.74	2.1	1.38
	10.56	10.5	9.42	9.36	10.32	10.26	1.32	1.8
	9.48	9.36	8.76	8.7	9.48	9.36	1.86	1.2
	9.9	9.78	8.88	8.82	9.42	9.36	1.26	1.44
11	12.9	12.84	11.7	11.58	11.88	11.82	1.2	0.72
	12.6	12.48	11.28	11.16	11.34	11.22	1.44	0.78
	11.28	11.16	9.9	10.02	10.74	10.68	0.72	1.26
	10.32	10.14	9.24	9.42	10.14	10.02	1.5	1.08
	10.5	10.38	9.18	9.12	10.2	10.14	0.84	0.96
12	13.56	13.38	12.3	12.06	12.72	12.6	0.72	0.42
	13.14	13.02	11.82	11.76	12.48	12.3	0.84	0.18
	12.36	12.24	11.28	11.16	11.76	11.58	0.24	0.78
	10.68	10.38	10.62	10.5	11.16	11.1	0.78	0.78
	10.86	10.74	10.14	10.08	11.1	11.04	0.6	0.66

13	14.58	14.4	13.14	13.08	13.5	13.38	0.3	0.12
	13.92	13.8	13.02	12.96	13.08	13.02	0	0
	12.9	12.84	11.82	11.7	12.78	12.66	0.12	0.3
	11.64	11.52	11.4	11.34	12.3	12.18	0.3	0.3
	11.58	11.7	11.22	11.1	11.64	11.34	0.18	0.18
14	14.94	14.82	13.68	13.5	14.52	14.28	0.12	0.12
	14.28	14.22	13.62	13.56	13.98	13.86	0	0
	13.92	13.62	12.42	12.36	13.44	13.26	0	0
	12.84	12.78	13.02	12.96	12.96	12.72	0.12	0.06
	12.54	12.3	12.54	12.24	12.54	12.42	0	0
15	15.24	15.18	14.76	14.58	15.24	15.3	0	0
	14.76	14.76	14.7	14.58	14.4	14.34	0	0
	14.22	14.1	13.44	13.08	13.68	13.56	0	0
	13.38	13.32	14.1	13.74	13.5	13.44	0	0
	13.38	13.38	12.9	12.66	13.26	13.2	0	0

Supplementary Methods

Synthesis of tissue phantom[1]. 3 ml Tris-buffered saline containing csUCNP@C (0.5 mg ml^{-1}) was mixed with a certain amount of sodium azide and gelatin to a final concentration of 15 mM and 10 w/v%, respectively. Then the solution was heated to 50°C under constant stirring. When the gelatin was totally dissolved, the mixture was kept at 37°C. Hemoglobin and intralipid were added to the system to a concentration of 50 μM and 1 v/v%. Finally, the mixed solution was poured into a quartz cuvette for subsequent UCL spectra acquisition. As reported in the literature, the absorption coefficient (m_a) and the scattering coefficient (m_s) at 700 nm were approximately 1.0 mm^{-1} and 0.5 mm^{-1}, respectively[1]. This gelatin-based phantom mimicked physiological tissue and represented a good physiological model for *in vivo* tissue.

Histological analysis. Two days after photothermal treatment, HeLa cell tumor-bearing nude mice from the treatment group and control group were sacrificed. Tumor tissues and major organs from these mice were harvested, fixed in 5% polyoxymethylene solution, routinely processed in paraffin, sectioned at 8 μm, stained with hematoxylin and eosin (H&E), and observed using a digital microscope. Examined tissues included liver, spleen, kidney, heart, lung and tumor.

Supplementary Reference

1. De Grand, A.M., *et al.* Tissue-like phantoms for near-infrared fluorescence imaging system assessment and the training of surgeons. *J. Biomed. Opt.* **11**, 014007 (2006).
